# Quantitative cytogenetics reveals molecular stoichiometry and longitudinal organization of meiotic chromosome axes and loops

**DOI:** 10.1371/journal.pbio.3000817

**Published:** 2020-08-19

**Authors:** Alexander Woglar, Kei Yamaya, Baptiste Roelens, Alistair Boettiger, Simone Köhler, Anne M. Villeneuve

**Affiliations:** 1 Department of Developmental Biology, Stanford University School of Medicine, Stanford, California, United States of America; 2 European Molecular Biology Laboratory, Heidelberg, Heidelberg, Germany; 3 Department of Genetics, Stanford University School of Medicine, Stanford, California, United States of America; Memorial Sloan-Kettering Cancer Center, UNITED STATES

## Abstract

During meiosis, chromosomes adopt a specialized organization involving assembly of a cohesin-based axis along their lengths, with DNA loops emanating from this axis. We applied novel, quantitative, and widely applicable cytogenetic strategies to elucidate the molecular bases of this organization using *Caenorhabditis elegans*. Analyses of wild-type (WT) chromosomes and de novo circular minichromosomes revealed that meiosis-specific HORMA-domain proteins assemble into cohorts in defined numbers and co-organize the axis together with 2 functionally distinct cohesin complexes (REC-8 and COH-3/4) in defined stoichiometry. We further found that REC-8 cohesins, which load during S phase and mediate sister-chromatid cohesion, usually occur as individual complexes, supporting a model wherein sister cohesion is mediated locally by a single cohesin ring. REC-8 complexes are interspersed in an alternating pattern with cohorts of axis-organizing COH-3/4 complexes (averaging 3 per cohort), which are insufficient to confer cohesion but can bind to individual chromatids, suggesting a mechanism to enable formation of asymmetric sister-chromatid loops. Indeed, immunofluorescence/fluorescence in situ hybridization (immuno-FISH) assays demonstrate frequent asymmetry in genomic content between the loops formed on sister chromatids. We discuss how features of chromosome axis/loop architecture inferred from our data can help to explain enigmatic, yet essential, aspects of the meiotic program.

## Abstract 2

This study introduces quantitative cytogenetic approaches for elucidating the spatial organization and molecular stoichiometry of components within biological macromolecular assemblies. These approaches are used to investigate how chromosome axes and their associated loops are organized during meiosis.

## Introduction

At the onset of meiotic prophase in organisms ranging from yeast to humans, newly replicated chromosomes adopt a highly specialized organization that enables diploid cells to produce haploid gametes. This reorganization involves the assembly of a discrete axial structure along the lengths of each conjoined sister-chromatid pair, with the majority of DNA organized into loops emanating from this axis. These meiotic chromosome axes and/or their constituent components contribute to virtually all major aspects of meiotic prophase, including chromosome movement, homologous chromosome pairing, formation of DNA double strand breaks (DSBs), assembly of synaptonemal complex (SC) between aligned homologs, and repair of DSBs by interhomolog recombination to yield crossovers that will ensure homolog segregation. Thus, understanding the structure and function of meiotic chromosome axes is crucial for elucidating the mechanisms responsible for faithful inheritance of chromosomes during sexual reproduction.

A combination of genetic, cytological, and biochemical approaches has identified the major components of meiotic chromosome axes (which are classically referred to as axial elements prior to synapsis and lateral elements in the context of fully formed SC) in multiple different experimental systems. The foundation of the chromosome axis is built up by meiosis-specific cohesin complexes that are composed of a mixture of canonical subunits and meiosis-specific subunits. Most notably, meiosis-specific kleisin subunits have been identified in multiple organisms [1], including Rec8p in budding yeast [2], RAD21L and REC8 in mammals [3, 4], and COH-3, COH-4, and REC-8 in *Caenorhabditis elegans* [1, 5, 6]. At the onset of meiotic prophase, these cohesin complexes mediate by unknown mechanisms the recruitment of other meiosis-specific axis components. Some of these, including SYCP2/SYCP3 in mice [7, 8] and Red1p in budding yeast [9], have been designated as “axis core” proteins, because they play a role in recruitment of meiosis-specific HORMA-domain containing proteins (e.g., Hop1p in yeast [10] or HORMAD1/2 in mammals [11]; referred to as “HORMADs” from here on). The HORMADs share a structural organization in common with spindle-assembly checkpoint protein Mad2, which has a capacity to form complexes with other proteins through topological entrapment of peptides known as “closure motifs” by a Mad2 domain known as a “safety belt” [12]. Recent work has demonstrated that yeast Red1p and mammalian SYCP-2/SYCP-3 each contain a single closure motif that mediates interactions with their respective HORMAD partners; further, Red1p homotetramers and SYCP2/SYCP3 heterotetramers are capable of forming oligomeric filaments in vitro [13]. In *C*. *elegans*, similar axis core components have not been identified, but 4 different HORMAD paralogs are present and constitute a hierarchical complex that builds up the meiotic chromosome axis (HTP-3, HTP-1/2, and HIM-3 [14–16]). Further, biochemical and in vivo experiments have demonstrated that HTP-3 recruits HTP-1/2 and HIM-3 to the axis by interacting with their HORMA domains via closure motifs located in its C-terminal tail [17]. Another recent study provided information regarding the relative cross-sectional positioning of cohesins and HORMADs within the context of the mature SCs of *C*. *elegans*, showing that HORMADs are located closer to the central region of the SC, whereas cohesin complexes are located closer to the bulk of the chromatin [18].

Although there has been substantial progress in identifying components of the meiotic chromosome axis and interactions among many of these components, much remains to be learned regarding how these proteins and interactions become organized along the length of chromosomes into a functional composite structure that mediates and coordinates key meiotic events. Ensemble/population-based measurements have identified preferential sites of association of multiple meiotic axis proteins in budding and fission yeast [19, 20], but these analyses do not address how many (and which) sites are occupied at the same time on individual meiotic chromosomes and in a given individual meiocyte. Further, a recent Hi-C-based study of mouse spermatocytes suggests a lack of reproducible loop positions during mouse meiosis [21]. It is also not known (either in meiotic cells or mitotically dividing cells) how many cohesin complexes are required to locally provide sister-chromatid cohesion (“single/simple ring” versus “handcuff model”, e.g., see [22]) or how many cohesins and other axis forming proteins load onto a given stretch of DNA and organize a pair of sister chromatids into a linear axis with emanating chromatin loops, as they appear in cytological preparations. Consequently, we currently lack understanding regarding how meiotic chromosome organization accomplishes certain essential tasks. For example, programmed meiotic DSBs must use the homologous chromosome rather than the sister chromatid as a repair template for recombinational repair, because it is necessary to form a crossover between homologs to provide the basis for a temporary link between the homologs that will ensure their correct segregation at the first meiotic division. An “inter-sister block” or “inter-homolog bias” favoring use of the homolog as a recombination partner is a long-known phenomenon in the meiotic recombination program and depends on chromosome axis proteins [23]. A pathway contributing to interhomolog bias via inhibition of Rad51 activity has been identified in budding yeast [24], but a key component of this pathway is not conserved. Thus, overall, how the interhomolog bias is achieved is not well understood on a mechanistic level. It is clear that our knowledge regarding how meiotic chromosome structures confer characteristic properties of the meiotic program would benefit from a fuller understanding of how the axis itself is organized.

**Fig 1:**
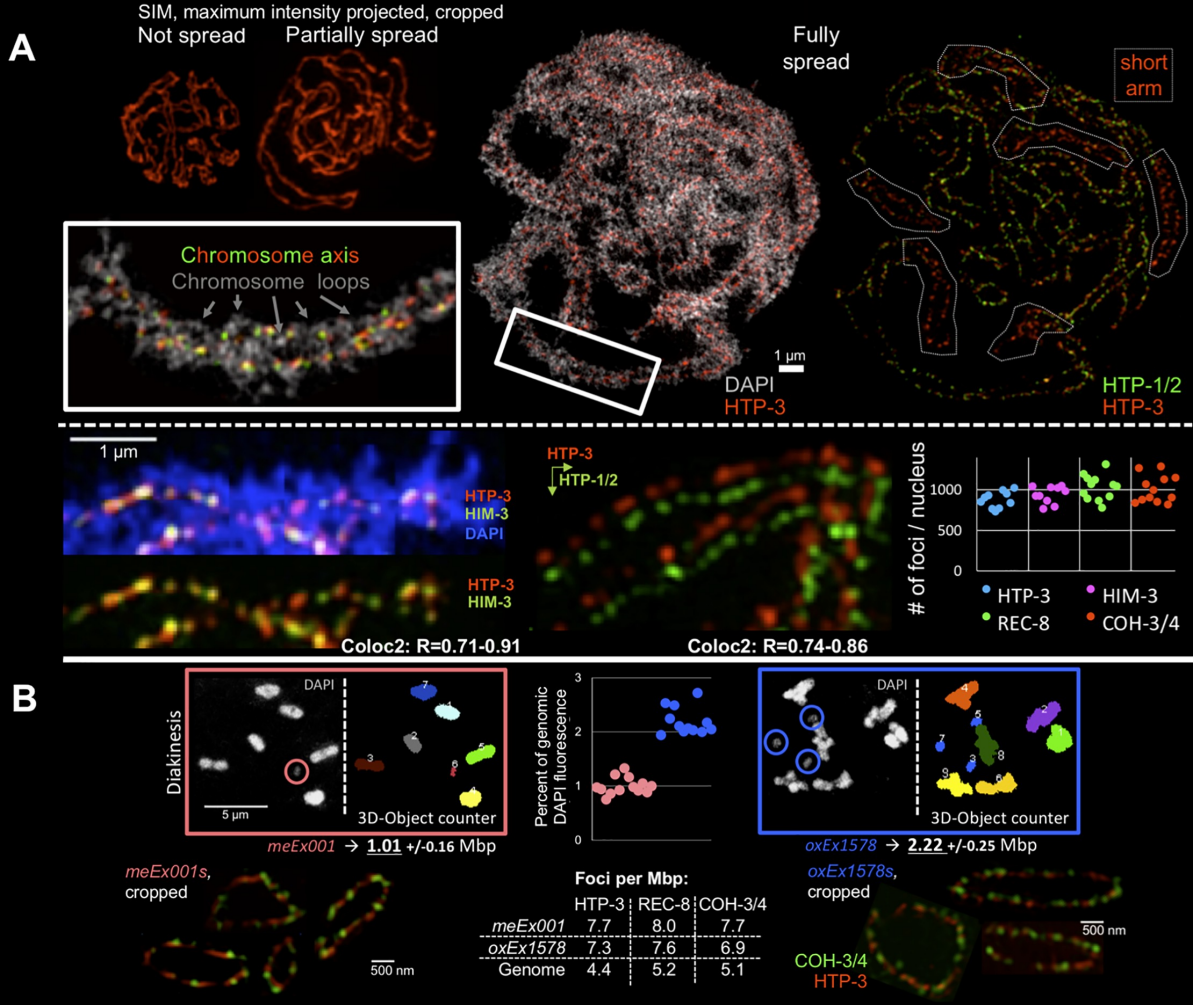
Components of the chromosome axis can be resolved as individual foci. (A, Top) Comparison of SIM images of in situ, partially spread, and fully spread individual meiotic prophase nuclei, displayed at the same magnification. In the image at the right, dashed lines outline the domains of each of the 6 bivalents where HTP-1/2 proteins have become depleted from the chromosome axis, indicating that this nucleus is at the late pachytene stage. (Bottom left, center) Images of cropped segments of synapsed chromosome pairs, illustrating both the pearls-on-a-string appearance of chromosome axis foci and colocalization of HTP-3 and HIM-3 (overlaid) or HTP-1/2 (images offset in both x and y); range of R values from ImageJ Coloc2 analysis for 8 to 10 individual fully spread nuclei. (Bottom, right) Quantification of numbers of HTP-3, HIM-3, REC-8, and COH-3/4 foci in individual fully spread nuclei; each data point represents a nucleus; plotted values are available in S1 Data. (B, Top) Method used to estimate the total DNA content of 2 distinct ExChrs, *meEx001* and *oxEx1578*. Sample deconvolved wide-field images of DAPI-stained chromosome complements from individual oocyte nuclei (with the ExChrs circled) are presented together with masks generated using the “3D Object Counter” ImageJ plugin; in the accompanying graph, each data point represents the total DAPI fluorescence for a single ExChr (2C), normalized to one-half of the total DAPI fluorescence measured for all 6 bivalents (4C) in the same nucleus. (Bottom, left and right) Individually cropped examples of ExChr *meEx001* (left) and *oxEx1578* (right) from fully spread nuclei stained for COH-3/4 or REC-8 together with HTP-3. (Bottom, center) Table representing the average densities per Mbp of HTP-3, REC-8, and COH-3/4 foci derived from our analyses counting foci for the whole genome and for the ExChrs. ExChr, extrachromosomal array; SIM, structured illumination microscopy.

Here, we report an analysis of the molecular stoichiometry and longitudinal architecture of the meiotic chromosome axis, using the well-established meiotic model organism *C*. *elegans*. Our work builds on and exploits a well-recognized feature of meiotic chromosomes from various model (and nonmodel) systems, namely, that when meiotic nuclei are strongly spread out in 2D on a glass slide (“fully spread” from here on), the continuous axis configuration (observed in in situ preparations and under mild/partial spreading conditions) is “spaced out,” and cohesins, axis core proteins, and/or HORMADs can appear as linear arrays of foci visible by electron microscopy or immunofluorescence (e.g., see the works by Smith and Roeder, Anderson and colleagues, Ishiguro and colleagues, and Kim and colleagues [9, 25–27] and Fig 1A). By combining this spreading approach with novel quantitative strategies and/or structured illumination microscopy (SIM), we reveal previously unrecognized features of meiotic chromosome organization. We demonstrate that different HORMAD proteins assemble into cohorts of defined numbers and co-organize the chromosome axis together with 2 structurally and functionally distinct cohesin complexes in defined stoichiometry. We show that half of the REC-8-containing cohesin complexes loaded during S phase are abruptly lost concurrent with loading of COH-3/4 cohesin complexes and axis assembly. In the resulting axis, individual cohesion-mediating REC-8 complexes occur on average every 130 to 200 kbp, interspersed with cohorts containing an average of 3 axis-organizing COH-3/4 complexes and 3 HORMAD complexes. Finally, we provide evidence that the axis architecture deduced from our analysis is associated with an inherent asymmetry in size, number, and genomic composition between the loops formed on sister chromatids. Together, our analyses provide a quantitative framework that will inform and constrain future experiments and models regarding meiotic chromosome axis organization and function. Moreover, the quantitative cytogenetic strategies applied here should be broadly applicable for investigating molecular stoichiometry in the context of other cellular structures and processes.

## Results and Discussion

### The meiotic chromosome axis is a linear array of discrete cohorts of cohesins and HORMADs

In contrast to the more continuous appearance of HORMAD signals observed in in situ preparations of *C*. *elegans* gonads and under partial spreading conditions (which maintain the spatial-temporal organization of the gonad largely intact), we find that in fully spread *C*. *elegans* meiotic prophase nuclei, the chromosome axis is resolved as individual HORMAD foci, arranged like pearls on a string (Fig 1A, S1A Fig), as observed in other organisms. The 3 types of HORMAD proteins that constitute the *C*. *elegans* meiotic chromosome axis (HTP-3, HTP-1/2, and HIM-3 [14–16]) colocalize together in most of these foci in early prophase nuclei when examined by SIM (Fig 1A), consistent with the demonstrated direct physical interactions among these components [15, 17]. Further, previously reported findings concerning distinct behaviors of different HORMAD proteins could be recapitulated in these fully spread preparations. For example, in late prophase, crossover designation triggers the reorganization of the *C*. *elegans* meiotic bivalent into 2 distinct domains: a long arm, where all the HORMADs are present, and a short arm, where only HTP-3 and HIM-3 are present [16]; this reorganization is clearly visible in the fully spread preparations (Fig 1A). We count approximately 1,000 HORMAD-containing foci (HIM-3 and HTP-3) in fully spread pachytene nuclei (Fig 1A, bottom right).

*C*. *elegans* HORMAD protein HTP-3, which is required to load all the other HORMADs [15], appears to play a role in axis organization analogous to the axis core proteins (Red1 and SYCP2 and 3) identified in other organisms. In the absence of HTP-3, meiotic cohesin complexes still bind chromatin and hold sister chromatids together until anaphase [6], but they are not arranged as a continuous axis or in a pearls-on-a-string-like configuration during prophase (S1 Fig). Thus, absence of HTP-3 phenocopies budding yeast *red1* mutants (in which axes do not form but cohesin binding is unaffected) rather than *hop1* mutants (in which Red1p and Rec8p containing axes form) [2, 28]. Therefore, we speculate that HTP-3, which contains 6 closure motifs that mediate associations with HIM-3 and HTP-1/2 [17], functions as the axis core component in *C*. *elegans*.

During metazoan meiosis, chromosome axis organization and meiotic sister-chromatid cohesion are mediated by at least 2 different cohesin complexes that contain distinct kleisin subunits and/or load at distinct times. Sister-chromatid cohesion is mediated by complexes containing REC-8 in worms, REC8 in mammals, and C(2)M in flies, whereas axis formation requires COH-3 and COH-4 in worms, REC8 and RAD21L in mammals, and the SOLO-SUNN-containing cohesin complex in flies [5, 6, 29–31]. Because COH-3 and COH-4 are products of a recent gene duplication, are functionally redundant, and are recognized by the same antibody, they are referred to as COH-3/4 from here on [6, 32]. We count similar numbers of foci for both REC-8 cohesin complexes and COH-3/4 cohesin complexes as we count for HORMAD protein foci (about 1,000 foci per nucleus; Fig 1A). If we consider the numbers of HORMAD foci, REC-8 foci, or COH-3/4 foci to be distributed along the diploid genome (2 × 100 Mbp), we can estimate an average of one focus (of each type) approximately every 200 kbp.

Because overlap between chromosome segments in spreads of whole nuclei might result in an underestimate of foci numbers, we sought to validate this estimate by measuring numbers of foci on well-separated DNA segments of defined size. Our approach was to evaluate numbers of cohesin and HORMAD foci on extrachromosomal arrays (ExChrs), which are minichromosomes that form via fusion of linear or circular DNA of any origin upon injection into the *C*. *elegans* gonad. ExChrs are visible as additional DAPI bodies in oocytes, which allows an approximation of their size (Fig 1B). Depending on the mixture of injected DNAs, ExChrs can be highly repetitive or complex in DNA sequence. Repetitive ExChrs acquire heterochromatic marks and are silenced in the germ line, whereas complex ExChrs can be transcriptionally active in germ cell nuclei [33]. ExChrs can be transmitted through mitosis and can be inherited across generations, albeit in a non-Mendelian fashion [34, 35].

Here, we analyzed ExChr *meEx001*, a complex array that has 95% *Escherichia coli* genomic DNA, and *oxEx1578*, a repetitive array that has 0% *E*. *coli* genomic DNA. Based on 3D DAPI intensity measurements in diakinesis-stage oocytes, we estimate that *meEx001* contains about 1 Mbp of DNA and *oxEx1578* contains about 2.2 Mbp, corresponding to approximately 1% and 2% of the size of the *C*. *elegans* haploid genome, respectively (Fig 1B). Interestingly, we found that ExChrs are ring chromosomes (S2A Fig) as suggested by S.K. Kim in 1992 (http://wbg.wormbook.org/wli/wbg12.2p22). Both *meEx001* and *oxEx1578* form chromosome axes during meiotic prophase but do not engage in synapsis when more than one ExChr is present in the nucleus (S2B Fig).

In fully spread nuclei, we find that ExChrs are decorated by HTP-3, COH-3/4, and REC-8 foci in a pattern similar to that observed on normal chromosomes (Fig 1B). Numbers of HORMAD foci and cohesin foci on a given ExChr are strongly correlated (S2B Fig). Further, the average numbers of foci observed for these 2 ExChrs were proportional to the amount of DNA present, as we detected twice the number of foci on the ExChr that contains twice the amount of DNA (*meEx001*: HTP-3: 7.8 [±2.4] foci, REC-8: 8.1 [±2.5] foci, and COH-3/4: 7.8 [±2.6] foci; *oxEx1578*: HTP-3: 16.1 [±2.9] foci, REC-8: 16.8 [±2.8] foci, and COH-3/4: 15.3 [±3.1] foci; Fig 1B and S2B Fig).

Our observation that *meEx001* and *oxEx1578* exhibit the same average numbers of cohesin and HORMAD foci (approximately 1 focus of each type per 130 kbp of DNA) is interesting given that these 2 ExChrs share almost no sequences in common with each other or with the *C*. *elegans* genome (except for a promoter fragment and plasmid sequences in the selection markers that are present in vastly different amounts in the 2 ExChrs). Thus, we conclude that HORMAD, REC-8, and COH-3/4 foci occur in similar numbers along chromosomal DNA as a function of chromosomal size and that they can do so apparently independently of DNA sequence.

### Nonrandom arrangement of structurally and functionally distinct cohesin complexes along chromosome axes

Previous work provided evidence that REC-8 and COH-3/4 cohesin complexes play functionally distinct roles during *C*. *elegans* meiosis [6, 32]: REC-8 has been demonstrated to mediate sister-chromatid cohesion, whereas COH-3/4 plays a key role in axis organization (S3A Fig). Further, these functionally distinct complexes differ in their timing of association with chromatin: REC-8 cohesins (like murine REC8) load during premeiotic DNA replication, whereas COH-3/4 cohesins (like murine RAD21L) load after completion of S phase, at the beginning of prophase (for review: the work by Ishiguro [1]) and are insufficient to maintain connections between sister chromatids in the absence of recombination [29]. Our data support and extend these findings substantially in several ways.

**Fig 2:**
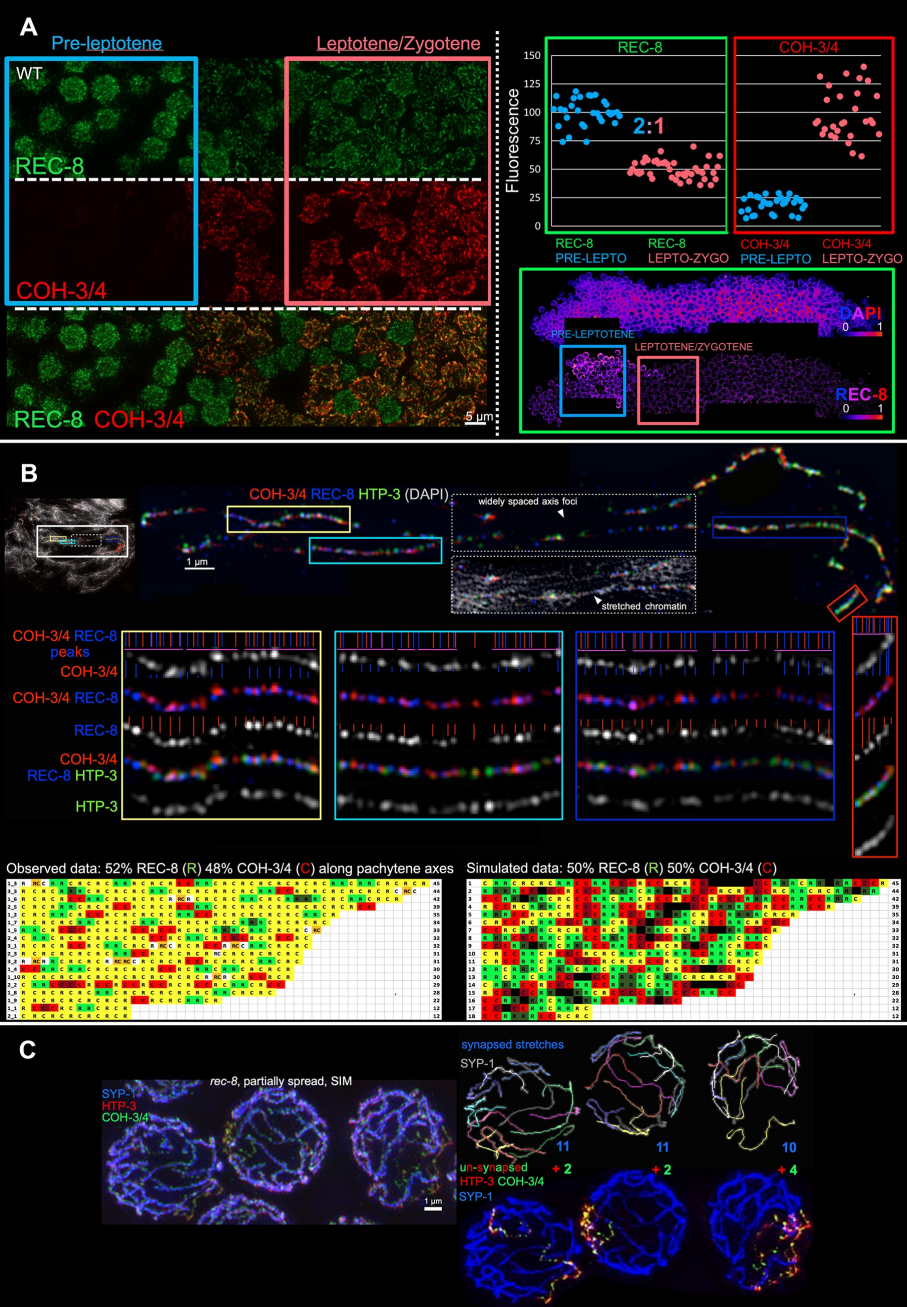
Nonrandom distribution of functionally and structurally distinct cohesin complexes. (A) Reduction in chromosome-associated REC-8 cohesion-conferring complexes occurs concurrently with axis formation. (Left) Deconvolved wide-field images of a partially spread gonad from a worm expressing REC-8::GFP, stained for GFP, COH-3/4, and DNA, illustrating that COH-3/4 becomes detectable on chromatin at leptotene entry and REC-8 signal intensity drops concurrently. As nuclei in this region of the gonad move approximately one row every 40 minutes [36], the fact that nuclei with medium levels of COH-3/4 or REC-8 are rarely observed suggests that this is a rapid transition. (Right) Quantitation of total REC-8 or COH-3/4 fluorescence in nuclei at preleptotene and leptotene/zygotene stages, measured using SUM projections of 3D-stiched gonads. Each data point represents a single nucleus; for each of the 4 gonads analyzed for each kleisin, fluorescence levels were normalized to 100 for the mean fluorescence in preleptotene nuclei for REC-8 and for the mean fluorescence in leptotene/zygotene nuclei for COH-3/4. REC-8 fluorescence drops at leptotene entry to half its preleptotene levels; plotted values are available in S1 Data. The images below the graph illustrate the drop in REC-8 using the indicated color scale to depict fluorescence signal intensity and boxes to indicate the regions of the gonad where fluorescence was quantified. (B) Nonrandom, alternating distribution of cohesin complexes containing different kleisin subunits along chromosome axes. (Top) SIM image of an individually cropped bivalent from a fully spread prophase nucleus, illustrating lack of colocalization of COH-3/4 and REC-8 signal peaks, even when axis segments are well preserved (colored boxes). The blue and red vertical lines in each enlarged segment represent the positions of REC-8 (blue) and COH-3/4 (red) signal peaks. Purple horizontal lines represent continuous stretches of alternating REC-8 and COH-3/4 signal peaks. (Bottom) Each line in the in the table represents a single, Z-projected, and straightened axis segment, with R indicating REC-8 foci and C indicating COH-3/4 foci. Yellow indicates that both direct neighbors of a given kleisin focus are of the other kleisin type. Red (COH-3/4) or green (REC-8) indicates that the indicated focus has one identical neighbor and one different neighbor. Darkening red or green represent more than 2 kleisins of the same kind in a row. Orange represents 2 nonresolvable REC-8 and COH-3/4 signal peaks (such as the first signal peak in the top, right). Axis segments are sorted by the number of cohesin foci (indicated at the right); all analyzed segments are presented (*n =* 18 segments from 3 nuclei). REC-8 and COH-3/4 foci alternate with each other in a highly nonrandom fashion (e.g., stretches of 3 or more of the same kleisin were observed much less frequently than expected for a random distribution (10 observed versus 131 expected; *p* < 0.00001, chi-square test, www.socscistatistics.com). The table on the right represents simulated data obtained using 50% C and 50% R as input for https://www.random.org/lists. (C) Evidence for association of COH-3/4 cohesin complexes with individual chromatids. (Right top) SIM image of SYP-1, HTP-3, and COH-3/4 immunostaining in 3 meiotic prophase nuclei from a *rec-8* mutant gonad; all 3 nuclei shown display a combination of SCs and unsynapsed chromosomes. (Middle) Tracings of individual SCs from the above nuclei; blue numbers indicate the total number of SCs in each nucleus. (Bottom) Nuclei with SCs pseudocolored in blue together with images of unsynapsed chromosome axes (marked with HTP-3 and COH-3/4); green numbers indicate the number of unsynapsed chromosome axes present in each individual nucleus. When unsynapsed chromosome stretches were detected in the *rec-8* mutant, they were consistently present in even numbers. Moreover, in nuclei where all SCs and unsynapsed axes could be reliably traced, we found that 2 × (the number of SCs) + (the number of unsynapsed axes) = 24, corresponding to the total number of chromatids present. Based on these numbers (and SC lengths measuring approximately 75%–80% the lengths of normal SCs; see S3C Fig), we infer that SCs form between sister-chromatid pairs in the *rec-8* mutant and that unsynapsed chromosome stretches correspond to individual chromatids. SC, synaptonemal complex; SIM, structured illumination microscopy.

First, measurement of fluorescence levels in immuno-stained gonads demonstrated that half of the REC-8 cohesin complexes loaded during S phase are removed from chromatin concurrently with loading of COH-3/4 cohesin complexes and coalescence of meiotic chromosome axes (Fig 2A). This suggests that a fundamental shift in the relationships between sister chromatids may occur upon entry into meiotic prophase. The sharp drop of REC-8 molecules upon meiotic prophase entry does not depend on the presence of COH-3/4, as it occurs even in a *coh-4 coh-3* mutant (S3B Fig). Cohesin release factor WAPL-1 may play a role in REC-8 removal, as a drop in REC-8 levels upon meiotic entry is not observed in a *wapl-1* mutant (S3B Fig); however, interpretation of this finding is complicated by the fact that COH-3/4 cohesins load prematurely at low levels during S phase in the *wapl-1* mutant [29], so REC-8 may not be loaded at normal levels in this mutant.

Second, analysis of SIM images of fully spread chromosome axes revealed that not only do COH-3/4 and REC-8 signal peaks usually not coincide, but COH-3/4 and REC-8 signal peaks usually occur in a largely alternating pattern along a given axis stretch (Fig 2B). This striking alternating arrangement is significantly different from simulated random positioning along a theoretical axis, strongly suggesting a functional biological basis underlying the observed pattern (Fig 2B). We note that although HORMAD protein foci were detected in numbers similar to the numbers of REC-8 and COH-3/4 foci (Fig 1), we did not detect a consistent longitudinal pattern in their arrangement relative to cohesins along the axis. However, HORMAD, REC-8, and COH-3/4 foci do occur in approximately equal proportions (1:1:1) locally along the chromosome axis; further, HORMAD foci can partially overlap with REC-8 foci, COH-3/4 foci, or both, albeit with their signal peaks often considerably offset (Fig 2B).

Third, analysis of spread nuclei from *rec-8* mutants provides additional evidence for a functional distinction between REC-8 and COH-3/4 cohesin complexes by demonstrating a capacity for COH-3/4 cohesin complexes to interact with individual chromatids. In *rec-8* mutants, when only COH-3/4 cohesin complexes are present on chromatin during meiotic prophase, SCs frequently form and crossover recombination apparently occurs between sister chromatids (rather than between homologs), suggesting that axes assemble along individual chromatids in this context [29],[37]. However, not all chromosomes engage in synapsis in *rec-8* mutants [37], and we were able to trace the paths of both synapsed and unsynapsed axes in late prophase nuclei of *rec-8* mutants (Fig 2C). This analysis confirmed the interpretation that continuous chromosome axes (containing HTP-3 and COH-3/4) do indeed form along individual chromatids in the *rec-8* mutant (Fig 2C and S3C Fig). Thus, we infer that whereas REC-8 cohesin complexes are required to confer cohesion between sister chromatids, COH-3/4 cohesin complexes are sufficient to organize chromosome axes and, moreover, can topologically embrace or bind to single chromatids (rather than sister-chromatid pairs), at least when REC-8 cohesin is absent.

### Inferring the numbers and stoichiometry of cohesin and HORMAD molecules in chromosome axis foci

Next, we set out to determine the numbers of the different cohesin and HORMAD proteins present in the individual axis foci that we observe cytologically.

**Fig 3:**
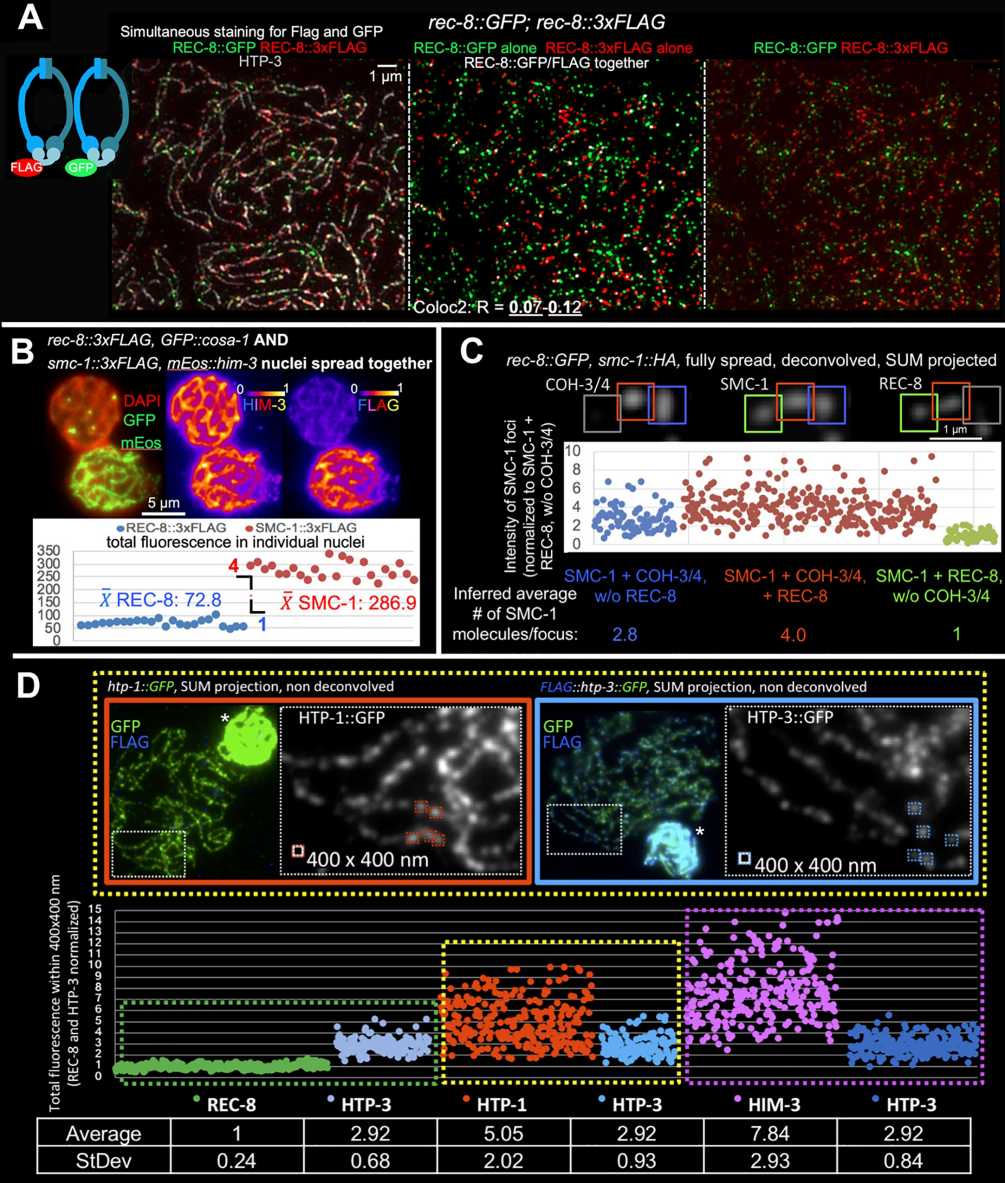
Quantitation of numbers of chromosome axis proteins present in individual axis foci. (A) Sample SIM images of fully spread nuclei from a worm expressing both REC-8::GFP and REC-8::3xFLAG, immunostained for HTP-3, FLAG, and GFP. All primary antibodies (anti-FLAG, ant-GFP, and anti-HTP-3) were applied at the same time. Middle panel was generated using ImageJ plugin “ColocThreshold,” with white indicating the infrequent pixels with significant FLAG and GFP colocalization (colocalizing pixels are in white; noncolocalizing pixels are in red and green); contrast and brightness were adjusted for display to improve visibility of the white pixels. Although GFP and FLAG signals both localize to chromosome axes (marked by HTP-3, left), they usually do not colocalize with each other. Coloc2 analysis using ImageJ confirmed the infrequent colocalization of FLAG and GFP by pixel intensity-based assessment (R(total) = 0.07–0.12; *n* = 28 nuclei). (B) Comparing the total amounts of chromatin-bound SMC-1 and REC-8. Gonads from worms expressing either SMC-1::3xFLAG and mEos::HIM-3 or REC-8::3xFLAG and GFP::COSA-1 were spread together on the same slides, immunostained for HIM-3 and FLAG, and imaged together using the same conditions. GFP::COSA-1 and mEos::HIM-3 served as internal markers to identify the genotypes of the nuclei being imaged; for the example nuclei depicted, similarities and difference in fluorescence intensities for HIM-3 and FLAG signals are illustrated using the indicated color scheme (generated using the Fire LUT from ImageJ). Graph indicates the total FLAG fluorescence measured in ROIs surrounding individual nuclei in SUM-intensity projections; each data point represents a single nucleus. For each genotype, 6 late pachytene nuclei each from 4 different gonads were assayed. All values obtained are displayed, with no normalization applied. The ratio of the average total fluorescence values is 1 REC-8::3xFLAG: 3.94 SMC-1::3xFLAG. (C) Inferred number of SMC-1 molecules in individual axis foci. The total fluorescence for SMC-1::HA, REC-8::GFP, and COH-3/4 was measured in individual 800 × 800 nm ROIs surrounding individual foci in SUM-intensity projections of fully spread nuclei; each data point represents the total fluorescence of SMC-1::HA in a single focus. As indicated in the cropped image on the top, SMC-1 foci were categorized as localizing with COH-3/4 but not REC-8 (blue, left in the graph); localizing with both COH-3/4 and REC-8 (red, middle in the graph); or localizing with REC-8 but not COH-3/4 (green, right). All values obtained are plotted in the graph on the bottom. The Y-axis (representing arbitrary fluorescence units) is normalized to the mean fluorescence of SMC-1 foci that only localize with a REC-8 focus but not with a COH-3/4 focus; this value is set to 1, because we consider REC-8 foci to represent single cohesin complexes. (D) Sample images (top) and quantitative data (graph and table) for experiments measuring fluorescence intensities of individual foci in parallel for pairwise combinations of axis proteins. Nuclei from worms expressing 3xFLAG::HTP-3::GFP and nuclei from worms expressing REC-8::GFP, HTP-1::GFP, or GFP::HIM-3 were spread together on the same slides, immunostained for GFP and FLAG, and imaged together using the same conditions. Presence or absence of FLAG staining (blue in the sample images) was used identify the genotype of the nuclei; asterisks indicate unspread nuclei of the same genotype that were present in the same field. Total fluorescence was measured in 400 × 400 nm ROIs surrounding well-separated individual foci in SUM-intensity projections; each data point represents a single focus. Different pairwise data sets were normalized to each other using the mean fluorescence of HTP-3::GFP foci as a normalization standard. The Y-axis (representing arbitrary fluorescence units) is normalized to mean REC-8::GFP = 1, because we consider REC-8 foci to represent single molecules. Plotted values for panels B, C, and D are available in S1 Data. ROI, region of interest; SIM, structured illumination microscopy.

First, we present evidence indicating that most individual REC-8 foci detected in fully spread nuclei represent single REC-8 cohesin complexes. We examined chromosome axes in fully spread meiotic chromosomes from worms expressing 2 differently tagged versions of REC-8 (REC-8::3xFLAG and REC-8::GFP) in the same animal; no untagged REC-8 was expressed in these worms. If REC-8 foci represented cohorts of multiple REC-8 cohesin complexes, we would have expected to observe frequent colocalization of the 2 tags. Instead, visualization by immunofluorescence and SIM imaging revealed that both tagged proteins are integrated into the chromosome axes, but the signals corresponding to the 2 tags usually do not colocalize (Fig 3A). Importantly, the same result was obtained when detection of FLAG and GFP was performed sequentially, in either order, indicating that lack of colocalization of the 2 tags was unlikely to have been caused by antibody interference (S4A Fig).

We also employed an independent approach, using purified GFP protein as a single-molecule standard, to evaluate the numbers of REC-8::GFP molecules present in meiotic axis foci (S4B Fig). Specifically, we spotted purified native GFP protein molecules on positively charged slides, and we spread and fixed nuclei from worms that express REC-8::GFP (as the sole source of REC-8 protein) on top of them. We then compared the immunofluorescence signals associated with individual GFP protein molecules with those of REC-8::GFP axis foci, visualized either with monoclonal or polyclonal antibodies against GFP. We found that measured immunofluorescence signals for REC-8::GFP cohesin foci on chromosomes were similar to those measured for the individual GFP molecules (1.2:1).

Based on these analyses, we conclude that most of the individual REC-8 foci in the fully spread preparations each represents a single cohesin complex. We note that REC-8-containing cohesin complexes appear to be the only cohesins that connect sister chromatids during meiotic prophase in a classical cohesive manner, as COH-3/4 complexes not only bind to chromosomes after replication but bind to individual chromatids and are insufficient to mediate sister-chromatid cohesion in the absence of REC-8 (Fig 2C [29, 32, 37]). Thus, the fact that REC-8 (cohesive) cohesins usually occur locally as individual complexes is not consistent with the handcuff model for sister-chromatid cohesion, which requires pairs of complexes to act together at any given site. Instead, our data strongly support the single ring hypothesis wherein sister-chromatid cohesion is mediated locally by topological entrapment of 2 sister chromatids by a single cohesin ring.

Having established that single cytological REC-8 foci usually represent single REC-8 molecules, we set out to deduce the numbers and relative stoichiometry of other cohesin subunits and different HORMAD components associated with meiotic chromosome axes using quantitative analysis of wide-field immunofluorescence images.

We used 2 different independent approaches to infer the relative numbers of REC-8 and COH-3/4 containing cohesin complexes. First, we measured and compared the total amount of chromatin-bound SMC-1 (which is present in all cohesion complexes) and chromatin-bound REC-8. Nuclei from gonads of 2 distinct genotypes, one expressing REC-8::FLAG (identified by GFP::COSA-1 expression) and the other expressing SMC-1::FLAG (identified by mEos::HIM-3 expression), were spread together on the same glass slide, immunostained for the Flag epitope, GFP (to detect GFP::COSA-1 and mEos::HIM-3), and HIM-3, and imaged using the same conditions (Fig 3B). Whereas anti-HIM-3 and DAPI fluorescence were not distinguishable between the 2 genotypes, the average total FLAG immunofluorescence signal for the nuclei expressing SMC-1::FLAG was 4 times that for the nuclei expressing REC-8::FLAG. Thus, we estimated there to be 4 times more chromatin/DNA bound SMC-1 than REC-8 molecules. We corroborated and extended this finding using an orthogonal approach in which we visualized and measured SMC-1 fluorescence signals (visualized with an HA tag) in 800 nm × 800 nm ROIs encompassing individual SMC-1 foci (Fig 3C). SMC-1 fluorescence values were compared for 3 classes of foci, i.e., those associated with (a) COH-3/4 but not REC-8, (b) both COH-3/4 and REC-8, (c) REC-8 but not COH-3/4. The average SMC-1::HA fluorescence measured for “COH-3/4 only” foci was 2.8× that measured for “REC-8 only” foci, and the average SMC-1::HA fluorescence for SMC-1 foci associated with both COH-3/4 and REC-8 was 4× that for “REC-8 only” foci. Further, the total fluorescence measurements for COH-3/4 and for SMC-1 within the same foci are highly correlated (S4C Fig), reinforcing the idea that REC-8 and COH-3/4 are the major, and possibly only, kleisins present in chromatin-bound cohesin complexes during meiosis [6]. In summary, our 2 different quantification approaches together revealed that whereas individual REC-8 foci each represent one cohesion-mediating cohesin complex, COH-3/4 foci represent, on average, 3 axis-organizing cohesin complexes.

We also wished to determine the number and relative stoichiometry of different HORMAD components in the chromosome axis foci. To this end, we made pairwise comparisons of relative fluorescence intensities of foci for different axis components marked with the same tag, using HTP-3 as a standard for aligning the different comparisons. For each of these experiments, we isolated nuclei from 2 different genotypes (one expressing 3xFLAG::HTP-3::GFP and one expressing REC-8::GFP, GFP::HIM-3, or HTP-1::GFP), then mixed and spread them together on the same slide, and stained and imaged them using the same conditions. For the data in Fig 3D, we measured fluorescence within 400 × 400 nm ROIs encompassing well-isolated individual foci and normalized our data to the mean fluorescence measured for REC-8 foci, because we consider these to represent single molecules. Using this approach, we estimated that a single cytological HORMAD focus contains, on average, 2.9 (±0.7) HTP-3 molecules, 5.1 (±2) HTP-1 molecules, and 7.8 (±2.9) HIM-3 molecules (Fig 3D). The relative ratios of REC-8::GFP to HTP-3::GFP (1:2.9) and HTP-3::GFP to GFP::HIM-3 (1:2.7) calculated using this approach are in good agreement with those estimated using a different method that evaluates total fluorescence in partially spread nuclei (1:3.6 for REC-8::GFP to HTP-3::GFP and 1:3.2 for HTP-3::GFP to GFP::HIM-3; S5A Fig).

HORMAD proteins HTP-1 and HTP-2 are nearly identical, encoded by the *htp-1* gene, which produces the bulk of the HTP-1/2 protein and is essential for successful meiosis, and the *htp-2* gene, which is largely dispensable for meiosis [16, 38, 39]. However, double mutant analyses indicate that protein derived from the *htp-*2 gene also contributes to axis function. Thus, the value calculated for HTP-1::GFP is expected to be an underestimate of the total number of HTP-1/2 molecules associated with meiotic chromosome axes in nuclei that contain a mixture of tagged HTP-1 and untagged HTP-2. Indeed, we found that the ratio of HTP-1::GFP to HTP-3::GFP was significantly higher for nuclei that lacked HTP-2 (1.9:1) than for nuclei where HTP-2 was present (1.4:1; S5B Fig). Thus, we infer that a single HORMAD focus most likely contains an average of 6 HTP-1/2 molecules during wild-type (WT) prophase.

**Fig 4:**
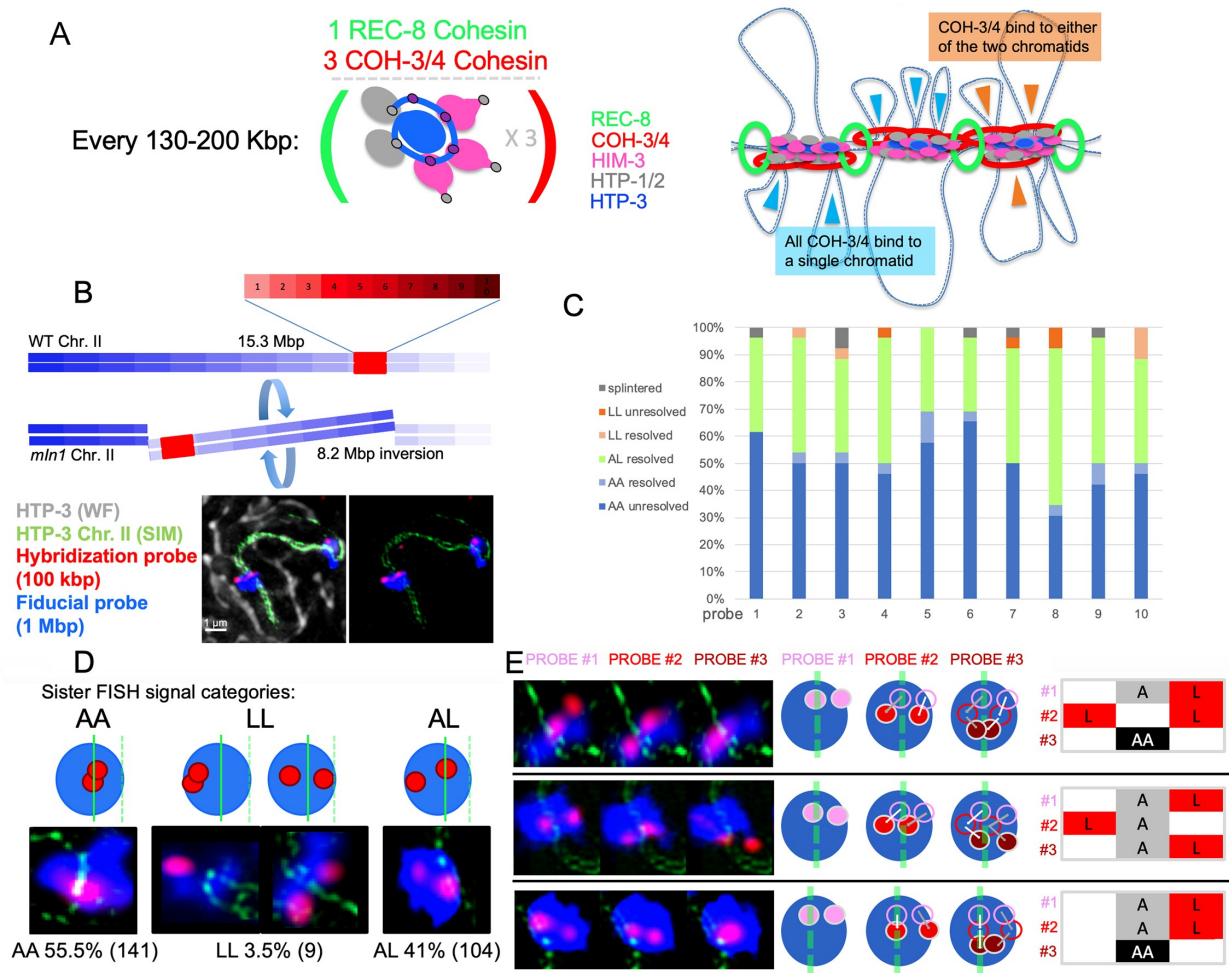
Model for chromosome axis organization and evidence for asymmetry between sister-chromatid loops. (A, left) A quantitative model for chromosome axis foci composition, stoichiometry, and density derived from the data presented here and in the work by Kim and colleagues [17]. (Right) Model of chromosome axis organization based on the data presented; see text for description. (B) Schematic illustrating the use of *mIn1*, a rearranged version of chromosome II harboring an 8.2 Mbp internal inversion, for immuno-FISH experiments assessing sister-chromatid loop relationships. *mIn1* heterozygosity results in wide (>7 Mbp) separation between the FISH signals for the normal and rearranged homologs, thereby enabling assessment of sister-chromatid signals. The red bar represents the 1 Mbp region covered by 10 individual 100 kbp oligopaint FISH probes, which were detected sequentially. In the example image shown, a projected 3D-SIM image of the HTP-3 signals (green), corresponding to the individually cropped chromosome II bivalent from an *mIn1/+* heterozygous nucleus, is overlaid on the wide-field image of HTP-3 (gray) for the whole nucleus. FISH signals from the 1 Mbp fiducial probe are depicted in blue, and the 100 kbp detection probe for the first hybridization step are depicted in red. Note that the *mIn1* chromosome harbors an insertion within the inverted segment and therefore displays a longer axis than the WT chromosome *II*, with which it is engaging in synapsis. (C) Stacked bar graph representing the frequency of occurrence of the indicated categories of sister-chromatid pair FISH signals for each of the 10 consecutive individual 100 kbp oligopaint FISH probes assayed (1–10). (A, axis associated; L, loop.) Note that in >90% of cases where only A signals were observed, the 2 sister signals were not resolvable from each other. This is consistent with sister chromatids usually being held at least roughly in register at REC-8-mediated cohesion sites; however, given the 100 kbp resolution of the FISH assay, it is also possible that nearby but nonidentical sister-chromatid sequences might be juxtaposed at individual REC-8 sites, potentially resulting in different lengths of DNA between adjacent REC-8 molecules for the 2 sister chromatids. Plotted data are available in S1 Data. (D, top) Schematic representation of categories of images observed for individual 100 kbp FISH probes. For each case, red circles indicate hybridization signals for an individual 100 kbp region, a blue oval indicates the hybridization signal for a fiducial probe corresponding to the entire 1 Mbp chromosome segment represented, and the solid green line indicates the chromosome axis corresponding to the hybridized chromosome. (The dashed green line indicates the axis corresponding to the synapsed partner chromosome, which is visible in some images but does not contain homologous DNA sequences at the same position owing to heterozygosity for a chromosomal inversion.) (Bottom) Example images illustrating the individual categories of sister FISH signals schematized above, with the observed incidence of each type indicated below (*n =* 254; the 6/260 cases where FISH signals were splintered/diffuse were excluded from this analysis). (E, left) Example images illustrating 3 consecutive sequential hybridization steps detected using consecutive 100 kbp probes. (Center) Cartoons illustrating the sequential hybridizations depicted. (Right) Corresponding schematic diagrams of the type used to codify and represent image galleries presented in S6 Fig. FISH, fluorescence in situ hybridization; SIM, structured illumination microscopy; WT,wild type.

The wide ranges of fluorescence values measured for HTP-1 and HIM-3 foci likely reflect biological variation in the numbers of HTP-1 or HIM-3 molecules present in individual HORMAD foci, as we find that HTP-1/2 and HIM-3 protein levels in meiotic nuclei increase over course of meiotic prophase (S5C Fig). In contrast, HTP-3 levels remain largely stable from meiotic entry through the end of pachytene (S5C Fig). Overall, our data are in strong agreement with biochemical and structural data from a recent study that investigated interactions among *C*. *elegans* HORMAD proteins [17]. This study found that the HIM-3 HORMA domain can bind to 4 of the 6 “closure” motifs in the C-terminal tail of HTP-3, and the HORMA domains of HTP-1/2 can bind to the remaining 2 motifs. Moreover, when the proteins are co-expressed in bacteria, HTP-3 and HIM-3 form complexes in a 1:2 or 1:3 ratio, whereas HTP-3 and HTP-1 do so in a 1:2 ratio. Thus, our data quantifying the numbers of foci per nucleus and their relative fluorescence are consistent with a model in which meiotic chromosome axes are assembled from interactions between individual cohorts of cohesin and HORMAD proteins in the following stoichiometry: [1 REC-8 cohesin]:[3 COH-3/4 cohesins]:[3 HTP-3: 6–9 HIM-3:6 HTP-1/2], distributed along chromosomes with an average density of one such “module” for every 130 to 200 kbp based on the number of individual REC-8-containing cohesive cohesin complexes (Fig 4A).

### Model for chromosome axis assembly and implications for axis/loop organization

Based on our data and findings from previously published literature [6, 17, 29, 32, 37], we propose the following model for the assembly and organization of meiotic chromosomes axes: Upon completion of premeiotic S phase and entry into meiotic prophase, half of the REC-8-containing cohesin complexes, which were loaded during S phase and hold sister chromatids together, are removed from chromatin by WAPL-1, resulting in connections between the 2 sister chromatids by a single REC-8 cohesin complex on average about every 130 to 200 kbp. Concurrently, COH-3/4-containing cohesin complexes bind and/or capture DNA of only one of the 2 sister chromatids, promoting the formation of loops in the intervals between the positions at which individual REC-8 complexes are maintaining cohesion between the sister chromatids. This organization is “locked in” by binding of a defined number of HTP-3 and other HORMADs, yielding an alternating occurrence of REC-8 cohesins and COH-3/4 cohesins along the resulting linear chromosome axis (Fig 4A).

When REC-8 is globally or locally absent, axis-promoting cohesin complexes (COH-3/4 in *C*. *elegans* or RAD21L in mice) and HORMADs assemble functional chromosome axes and loops on single DNA molecules (Fig 2C [37, 40]). Thus, we propose that on meiotic prophase chromosomes, COH-3/4-containing cohesin complexes also bind to individual chromatids between REC-8 mediated cohesion sites. In principle, the individual COH-3/4 complexes in each cytological focus (on average 3 complexes per cohort) could potentially bind to DNA from both sister chromatids (Fig 4A, orange arrowheads) or alternatively they could all bind in a coordinated manner to the same chromatid within any given interval between REC-8 cohesion sites (Fig 4A, blue arrowheads). The first possibility could result in the formation of either symmetric or asymmetric loops, depending on the number of COH-3/4 complexes present in a given cohort. The latter possibility predicts that DNA loops that form on the 2 sister chromatids between 2 REC-8 cohesion complexes would be inherently asymmetric: DNA on one sister chromatid would form a single long loop that is not bound by COH-3/4, whereas the DNA on the other sister chromatid would be more closely associated to the chromosome axis in several shorter loops that are organized by a cohort of COH-3/4 complexes.

### Evidence for frequent asymmetry of sister-chromatid loops

We used an iterative immuno-FISH (fluorescence in situ hybridization) approach [41] to investigate the organization of the chromosome loops associated with pairs of sister chromatids, both relative to each other and in relationship to the chromosome axis. Specifically, we sequentially detected and imaged pools of oligopaint FISH probes representing 10 consecutive 100-kbp regions spanning a 1-Mbp region of chromosome II on chromosomes prepared using partial spreading conditions. To be able to distinguish FISH signals from sister-chromatid pairs from signals derived from the homologous chromosome, we used worms heterozygous for an internal inversion of chromosome II (*mIn1*), which results in wide spacing between the FISH signals associated with the 2 homologs (Fig 4B). Conventional deconvolution microscopy images of HTP-3 axis signals were acquired in conjunction with acquisition of images for each FISH detection step, enabling registration of images corresponding to consecutive genomic positions. In addition, a 3D-SIM image of the HTP-3 signal was acquired and used to enable assessment of the spatial relationships between FISH signals and the axis (see Materials and methods).

Overall, most 100-kbp probe signals were detected either as a single unresolved focus associated with the chromosome axis (50%) or as 2 resolvable sister-chromatid foci (42%) with one or both sister signals clearly separated from the axis, i.e., in the loops (*n =* 260, Fig 4C); the remaining cases consisted of unresolved single signals present in loops (1.5%), pairs of resolved sister signals that were both axis-associated (4%), or signals that were fragmented and/or noncompact (2.3%). Analysis of these immuno-FISH data yielded 2 important insights regarding axis/loop organization.

First, our data indicate that there is no locus within the assayed 1 Mbp chromosome segment that is consistently associated with the chromosome axis during the pachytene stage. For each of the 10 probes, instances in which one or both sister signals were clearly resolved from the axis were observed in at least 27% of hybridizations (Fig 4C), indicating that none of the 100 kbp segments assayed contained DNA sequences that were consistently axis associated. Thus, we conclude that the genomic sequences associated with the chromosome axis are variable between different meiocytes.

Second, analysis of this data set revealed strong evidence for asymmetry between sister-chromatid loops (Fig 4D and 4E). The 100 kbp FISH signals that do not colocalize with the chromosome axis marker must represent DNA sequences that are located in chromosome loops (“L”). Conversely, FISH signals that correspond to axis-associated DNA or DNA in associated loops that are smaller than 100 kbp in size will colocalize with the axis marker (“A”). If sister-chromatid loops were consistently symmetric, we would expect that for any given 100 kbp DNA segment, sister FISH signals would typically either both be axis associated (AA) or both be in loops (LL), and cases in which one sister signal was axis associated and the other was in a loop (AL) would be rare. Our observed data clearly depart from this expectation (*p* < 0.00001), as 41% of sister FISH signal pairs were of the AL category and only 3.5% were of the LL category, with the rest being of the AA category (55.5%, *n =* 254 loci assayed, Fig 4D). At face value, the observed distribution of AA, AL, and LL signal pairs would appear to be compatible with that expected for independent behavior of the 2 sister chromatids (*p =* 0.1443). However, a different picture emerges when the expected behavior at sites of REC-8-mediated sister-chromatid cohesion is taken into consideration. Using the conservative estimate of an average density of one REC-8 cohesin complex every 200 kbp (Fig 1), half of the 100 kbp probes in any given hybridization sequence would be expected to include a REC-8-mediated cohesion site and thus would yield an “obligate” unresolved axis-associated signal in the immuno-FISH analysis. (This is based on sister chromatids usually being held at least roughly in register at REC-8 mediated cohesion sites, as indicated by our data; see Fig 4C.) If we remove the expected number of “obligate AA” sites (*n* = 127) from consideration when evaluating the behavior of sister-chromatid FISH signals, the new distribution of the remaining 127 signal pairs is 11% AA, 82% AL, and 7% LL; i.e., AL signal pairs (in which sister chromatids exhibit different behavior) now represent the preponderance of cases. This clearly differs from expectations for independent behavior of sister chromatids (*p* < 0.00001) and instead implies a strong propensity for sister chromatids to be asymmetric. Consistent with this interpretation, when we analyzed 39 sequences of consecutive FISH detection steps for individual chromosomes and considered the possible paths of loops that were >100 kbp in size (see S6 Fig and legend), we observed only a single case in which loops of the same size occurred at the same position on both chromatids (Fig 4E and S6 Fig).

As spreading of chromosomes and Z-projection of images might potentially introduce artifacts, we carried out a separate assay using a 200 kbp FISH probe in minimally disrupted tissue analyzed in 3D (S7 Fig). This approach corroborated our finding that the majority of chromosome loops are asymmetric. Specifically, we found that the majority of sister FISH signal pairs were in the AL class (57%), with only 5% in the LL class and the remainder (38%) in the AA category (*n =* 94 loci assayed, S7 Fig). Thus in summary, we conclude that the loops present on pairs of sister chromatids are frequently distinct from each other in size and/or position and, consequently, in genomic content. Indeed, our data are consistent with a model in which sister-chromatid loops are inherently asymmetric.

Asymmetry between sister-chromatid loops may be a widespread feature of meiotic prophase, as loop asymmetry was observed previously for giant loops of diplotene-stage lampbrush chromosomes of the newt [42]. Our observation of sister loop asymmetry during an earlier stage of meiotic prophase can potentially help to explain some important properties of the meiotic recombination program. Since the DSBs that serve as the initiating events of meiotic recombination are thought to be generated in loop regions that are tethered to the chromosome axis [20, 43], such asymmetry between sister-chromatid loops could potentially help to prevent DSBs from occurring at the same site on both sister chromatids during the same meiosis. Furthermore, the proposed axis-loop organization could also contribute to the interhomolog repair bias of meiotic recombination [44–46], as an intrinsic feature of the proposed configuration is that sister chromatids would be unlikely to share the same loop domains and thus would not be favored as DNA repair partners.

## Concluding remarks

The approaches employed in this work have enabled us to deduce the numbers, relative stoichiometry, and spatial distribution of multiple proteins that are central to the functional organization of chromosomes during meiosis. Further, our data regarding the number of cohesin foci and the stoichiometry of proteins within these foci can be used to calculate the density of cohesin complexes along *C*. *elegans* meiotic prophase chromosomes. Based on our evidence that most REC-8 foci represent single cohesin complexes and that COH-3/4 foci represent, on average, 3 cohesin complexes, we can infer that there are approximately 20 to 30 cohesin complexes per Mbp of chromosome length when each pair of sister chromatids is considered as a single conjoined entity. When considering the total amount of DNA in each sister-chromatid pair, this translates to a density of 10 to 15 cohesin complexes per Mbp of DNA. These measurements of cohesin complex density on *C*. *elegans* meiotic chromosomes are in remarkably close agreement with recent independent estimates of cohesin density deduced for mouse ES cells based on in-gel fluorescence measurements and fluorescence correlation spectroscopy (FCS)-calibrated imaging (5.3 complexes/Mb [47]) and for HeLa cells based on quantitative mass spectrometry and FCS-calibrated imaging (8.5–17 complexes/Mb [48]), supporting both the validity of our experimental approach and the broad relevance of our findings to the field of chromosome biology. Thus, we anticipate that other features revealed in the current work, such as mediation of cohesion by individual cohesin complexes, and spatial alternation between cohesion-mediating cohesin complexes and putative axis/loop-organizing cohesin complexes, may reflect generalizable principles and properties of chromosome organization that operate in other contexts. Finally, we note that the approaches that we devised here to quantify numbers and/or relative stoichiometry of chromosome axis components should be widely applicable for analyzing many other cellular structures and biological processes, enabling quantitative estimates of molecular components that can inform and constrain models regarding how such structures and processes assemble and function.

## Materials and methods

### *C*. *elegans* culture conditions

Worms were grown on *E*. *coli* (OP50) seeded NG agar plates at 20°C according to the standard method [49]. For experiments, worms were either selected as homozygous L4 larvae (from heterozygous strains maintained using balancer chromosomes) or were harvested as staged L1 larvae following bleaching of gravid adults (for strains maintained as homozygotes or for *mIn1/+* heterozygotes) according to the work by Stiernagle [49] and were analyzed 24 to 36 hours post L4 stage. For experiments using 2 tagged versions of REC-8, ATGSi23 (*rec-8*::*GFP; rec-8 (null)*) hermaphrodites were mated with CA1481 (*rec-8*::*3xFLAG*) males, and leptotene/zygotene nuclei of F1 heterozygous hermaphrodites, which expressed both REC-8 tagged proteins (and no untagged REC-8), were analyzed 36 hours post L4 stage.

### *C*. *elegans* strains used in this study

- N2
- ATGSi23: *fqSi23[Prec-8*::*rec-8*:::*GFP*::*rec-8 3’UTR + cb-unc-119(+)] II; rec-8(ok978) IV*
- CA1481: *mels8[Ppie-1*::*GFP*::*cosa-1 + cb-unc-119(+)] II; rec-8(ie35[rec-8*::*3xFLAG]) IV*
- TY4986: *htp-3(y428) ccls4251 I/hT2 (I*, *III)*
- ATG98: *wapl-1(tm1814) IV/nt1[qIs51] (IV*,*V)*
- TY5120: *+/nT1 IV; coh-4(tm1857) coh-3(gk112) V/nT1[qls51] (IV;V)*
- VC666: *rec-8(ok978) IV/nT1[qls51] (IV;V)*
- AV1079: *meEx001; rec-8(ok978)/nT1[qls51] (IV;V)*
- EG699: *ttTi5605 II; unc-119 (ed3); oxEx1578*
- CA1282: *him-3(ie114[gfp*::*him-3]) IV*
- CA1437: *htp-3(tm3655) I; ieSi62[Phtp-3*::*3×Flag*::*htp-3*::*GFP*::*htp-3 3′UTR + unc-119(+)] II; him-3(ie33[mEos2*::*him-3]) IV*
- ATGSi43: *fqSi37[htp-1*::*GFP cb-unc-119(+)] II; htp-1(gk174) IV*
- AV1080: *fqSi37[htp-1*::*GFP cb-unc-119(+)] II; htp-1(gk174) htp-2(tm2543) IV*
- AV1088: *smc-1(ie39[smc-1*::*intHA]) I; fqSi23[Prec-8*::*rec-8*:::*GFP*::*rec-8 3’UTR + cb-unc-119(+)] II; rec-8(ok978) IV*
- VC1474: *top-2(ok1930)/mIn1[mls14 dpy-10(e128)]* II
- CA1432: *smc-1(ie36[smc-1*::*3xFlag]) I; mels8[Ppie-1*::*GFP*::*cosa-1 + cb-unc-119(+)] II; him-3(ie33[mEos2*::*him-3]) IV*

### Microinjection of *C*. *elegans*

Microinjection was performed using a FemtoJet 4i injector system (Eppendorf) and an Axiovert 10 microscope (Zeiss). Injection mixes contained a total of 100 ng DNA in distilled water. The injection mix for *meEx001* contained the dominant selection plasmids pGH8 (*prab-3*::*mCherry*::*unc-54_3’UTR*, 2.5ng) and pBR186 (modified from pDD282, includes *sqt-1p*::*sqt-1(e1350)*:: *sqt-1_3’UTR _hsp16*::*Cre*::*tbb-2_3’UTR_rps-11p*::*HygR*::*unc-54_3’UTR*, 2.5ng) in addition to 95 ng *E*. *coli* (OP50) DNA, isolated by standard protocols, sonicated, and size selected for fragments of 1 to 5 kbp by agarose gel purification.

### Full spreading and staining of nuclei

Worms were staged by bleaching, grown at 20°C, and harvested 24 to 36 hours post L4 stage (pelleted by gravitation), washed 3 times in 0.35× nuclear purification buffer (NPB; 3.5 mM HEPES [pH 7.5], 14 mM NaCl, 31.5 mM KCl, 0.07 mM EDTA, 0.175 mM EGTA, 0.07 mM DTT, 0.035% Triton X-100). The resulting worm pellet was mixed with 1 volume 0.35× NBP and frozen as worm beads by dropping 50 μL drops of NPB-worm slurry into liquid nitrogen. A total of 2 to 10 beads were ground for 10 seconds using a liquid nitrogen cooled mortar and pestle. The ground tissue was transferred into a 50 mL Falcon with a kitchen spoon, thawed, and pipetted up and down several times to release nuclei. A total of 5 μL of this suspension was applied on an EtOH-washed 22 × 40 mm coverslip. A total of 50 μL of spreading solution (see below) was added, and nuclei were immediately distributed over the whole coverslip using a pipette tip. Coverslips were left to dry at room temperature (approximately 1 hour) and postdried for 2 more hours at 37°C, washed for 20 minutes in methanol at −20°C, and rehydrated by washing 3 times for 5 minutes in PBST. A 20-minute blocking in 1% w/v BSA in PBST at room temperature was followed by overnight incubation with primary antibodies at 4°C (antibodies diluted in 1% w/v BSA in PBST supplied with 0.05% w/v NaN_3_). Coverslips were washed 3 times for 5 minutes in PBST before secondary antibody incubation for 2 hours at room temperature. After PBST washes, the nuclei were immersed in Vectashield (Vector), and the coverslip was mounted on a slide and sealed with nail polish. Spreading solution: (for one coverslip, 50 μL): 32 μL of Fixative (4% w/v Paraformaldehyde and 3.2%–3.6% w/v Sucrose in water), 16 μL of Lipsol solution (1% v/v/ Lipsol in water), 2 μL of Sarcosyl solution (1% w/v of Sarcosyl in water).

### Partial spreading and staining of nuclei

Partial spreading of *C*. *elegans* gonads was performed as in the work by Pattabiraman and colleagues [50]. The gonads of 20–100 adult worms were dissected in 5 μL dissection solution (see below) on an EtOH-washed 22 × 40 mm coverslip. A total of 50 μL of spreading solution (see above) was added, and gonads were immediately distributed over the whole coverslip using a pipette tip. Coverslips were left to dry at room temperature (approximately 1 hour) and postdried for 2 more hours at 37°C, washed for 20 minutes in methanol at −20°C, and rehydrated by washing 3 times for 5 minutes in PBST. A 20-minute blocking in 1% w/v BSA in PBST at room temperature was followed by overnight incubation with primary antibodies at 4°C (antibodies diluted in 1% w/v BSA in PBST supplied with 0.05% w/v NaN_3_). Coverslips were washed 3 times for 5 minutes in PBST before secondary antibody incubation for 2 hours at room temperature. After PBST washes, the nuclei were immersed in Vectashield (Vector), and the coverslip was mounted on a slide and sealed with nail polish. Dissection solution: For Fig 1A (second nucleus on the top left), Fig 2A, Fig 4, S1B Fig, S2A Fig, S3A Fig, S5A Fig and S6 Fig, gonads were dissected in 10% to 30% v/v Hank’s Balanced Salt Solution (HBSS, Life Technology, 24020–117) with 0.1% v/v Tween-20. For Fig 2C, S2B Fig, S3C Fig and S7 Fig, gonads were dissected in 50% to 85% v/v HBSS (Life Technology, 24020–117) with 0.1% v/v Tween-20.

### Antibodies used in this study

The following primary antibodies were used: chicken anti-HTP-3 (1:500) [51], chicken anti-GFP (1:2,000; Abcam), mouse anti-GFP (1:500; Roche), rabbit anti-GFP (1:500) [52], mouse anti-HA (1:1,000; Covance/Biolegend), guinea pig anti-SYP-1 (1:200) [53], mouse anti-H3K9me2 (1:500; Abcam), rabbit anti-SMC-1 (1:200) [54], rabbit anti-COH-3/4 (1:5,000) [32], rabbit anti-REC-8 (1:10,000; Novus Biologicals), rabbit anti-HIM-3 (1:200) [14], rabbit anti-HTP-1/2 (1:500) [16], and mouse anti-FLAG (1:200; Sigma).

Secondary antibodies conjugated to Alexa dyes 405, 488, 555, or 647, obtained from Molecular Probes, were used at 1:500 dilution (Alexa 488 and 555), 1:200 (Alexa 647), and 1:100 (Alexa 405). In cases in which antibodies raised in mouse and guinea pig were used on the same sample, we used highly cross-absorbed goat anti-mouse secondary antibodies, obtained from Biotium (conjugated to CF488 or CF555, respectively) for secondary detection of the mouse primary antibody in order to avoid cross-reaction against antibodies raised in guinea pig.

### Imaging

Imaging, deconvolution, and 3D-SIM reconstruction were performed as in the work by Pattabiraman and colleagues [50]. Wide-field images were obtained as 200-nm spaced Z-stacks, using a 100× NA 1.40 objective on a DeltaVison OMX Blaze microscopy system, deconvolved and corrected for registration using SoftWoRx. Subsequently, gonads were assembled using the “Grid/Collection” plugin [55] in ImageJ. For display, pictures were projected using maximum intensity projection in ImageJ. 3D-SIM images were obtained as 125-nm spaced Z-stacks, using a 100× NA 1.40 objective on a DeltaVison OMX Blaze microscopy system, 3D-reconstructed and corrected for registration using SoftWoRx. For display, images were projected using maximum intensity projection in ImageJ or SoftWoRx. For display in figures, contrast and brightness were adjusted in individual color channels using ImageJ.

### Manual quantification of axis foci

Axis foci were counted manually on 32-bit Z-projected SIM images. For this analysis, foci are defined as fluorescence signals that (1) display individual maxima in nonoverlapping ROIs ≥ 3 × 3 pixels in size and (2) were at least 5 times brighter than background average (with most ranging from 10–100 times brighter than background).

### Assessing localization of REC-8 and COH-3/4 on straightened axes

Axis segments that did not twist around the homologous partner locally and were not spread on top of each other in maximum intensity Z-projected SIM images were manually traced and straightened using the ImageJ “Segmented Line Tool” and “Selection straighten” operation as a 10 pixel-wide band. Signal peaks in individual channels were manually identified and marked as a single pixel along the band. The individual channels were analyzed separately, and the outputs were overlaid prior to manual readout of the spatial relationships of peak positions, resulting in the graphs presented in Fig 2.

### Colocalization analysis using coloc2 and colocalization threshold

Colocalization was assessed by evaluating the correlation of pixel intensities over space using the Coloc2 plugin of ImageJ. R values around 0 indicated lack of colocalization, whereas an R value of 1 would indicate 100% colocation of signals in 2 channels, with their intensities completely correlated. 32-bit images were cropped and transformed into 8-bit single channels images. Background was subtracted in ImageJ using the sliding paraboloid background subtraction operation before running the Coloc2 or the Colocalization Threshold analyses.

### 3D-tracing of chromosomes

The 32-bit SIM images obtained from high salt spreads were transformed first into RGB color images and then into monochrome 8-bit images. In such images, individual chromosomes were traced using the “Simple Neurite Tracer” plugin of ImageJ [56]. For display and straightening of individual chromosomes, each individual chromosome path was exported as an 8-bit two-color mask with the “Fill Out” function of the “Simple Neurite Tracer.” This mask was used to crop the individual chromosome in 3D from an RGB stack. These pictures were projected in Z and straightened using ImageJ “Segmented Line Tool” and the “Selection straighten” operation.

### Comparing fluorescence of purified GFP protein and REC-8::GFP foci

Soluble native His-tagged EGFP protein, purified using a procedure that reliably yields monodisperse protein corresponding in size to monomeric EGFP, was obtained as a gift from Peter Jackson. The protein was diluted in PBS to a concentration of 3.3 nM; EGFP should be primarily monomeric under these conditions, as dimerization is strongly disfavored at this concentration. This diluted protein solution was applied to positively charged slides (“Superfrost Plus,” Fisher Scientific) and incubated for 30 minutes in a moist chamber. Most of the liquid was aspirated off, and, immediately afterward, nuclei isolated from worms expressing REC-8::GFP (in a *rec-8* deletion background; ATGSi23) were spread on top using the “Full spreading protocol” with no alterations.

GFP was visualized using rabbit polyclonal or mouse monoclonal anti-GFP primary antibodies and Alexa 647-labeled secondary antibodies (Molecular Probes/Invitrogen) directed against rabbit or mouse, respectively. In a sequential step, the chromosome axis was visualized by anti-HTP-3 and Alexa 555-labeled anti-chicken secondary (Molecular Probes/Invitrogen). Nondeconvolved wide-field images were SUM-intensity projected.

Fluorescence signals were measured for 4 × 4 pixel ROIs encompassing individual well-spaced REC-8::GFP foci or purified GFP protein foci from the same image. Background in the image was measured in same-sized ROIs, averaged, and subtracted. All obtained values are presented.

### Quantitation of relative levels of chromosome axis proteins present in individual axis foci

Nuclei from worms expressing 3xFLAG::HTP-3::GFP and nuclei from worms expressing REC-8::GFP, HTP-1::GFP, or GFP::HIM-3 were isolated in parallel, mixed, and spread together on the same slides. GFP was labeled using primary and secondary antibodies prior to primary and secondary detection of the FLAG epitope to identify the nuclei expressing 3xFLAG::HTP-3::GFP, and nuclei on the same slide were imaged in parallel using the same conditions. SUM-intensity projections were generated from nondeconvolved 32-bit images, and total fluorescence was measured in 400 × 400 nm ROIs surrounding well-separated individual foci. Fluorescence measurements were made for 25 to 70 well-separated foci per nucleus (4–9 nuclei for each axis component); each data point represents a single focus, and all data points measured are presented in the graphs. Different pairwise data sets were normalized to each other using the mean fluorescence of HTP-3::GFP foci as a normalization standard. The Y-axis (representing arbitrary fluorescence units) is normalized to mean REC-8::GFP = 1, because we consider REC-8 foci to represent single molecules.

### Whole-nucleus comparisons of anti-FLAG fluorescence

Gonads from worms expressing GFP::COSA-1 and REC-8::3xFLAG were dissected and mixed with gonads from worms expressing 3xFLAG::HTP-3::GFP and spread on the same slide using partial spreading. To avoid antibody exclusion artifacts, only the FLAG tag was detected by anti-FLAG immunostaining, and GFP was detected using native GFP fluorescence. Gonads were additionally stained with DAPI. Genotypes were distinguished by GFP::COSA-1 or HTP-3::GFP expression, respectively. Z-stacks were acquired using the same wide-field conditions and SUM-intensity projected without prior deconvolution. ROIs were drawn encircling late pachytene nuclei from 3 gonads for each genotype (20 nuclei per gonad). Total FLAG and DAPI fluorescence within each ROI were recorded; values presented in the graph are ratios between FLAG and DAPI fluorescence (to normalize for differences in numbers of Z planes occupied by the nuclei in the SUM projection and differences in ROI sizes). Values were further normalized to average value of REC-8::GFP = 1. All obtained values are presented.

### Design and generation of oligopaint FISH probes

Probes were designed to target a 1 Mbp region on chromosome II, between coordinates 11,500,001 and 12,500,001, as in the work by Mateo and colleagues [41]. The 1 Mbp region was divided into one hundred 10-kbp segments, and 100 probes were targeted to hybridize to each 10 kbp segment. Each probe consisted of (1) a unique nonoverlapping 40-nt sequence corresponding to genomic DNA, (2) a 20-nt barcode sequence identifying each 10 kbp segment, (3) a 20-nt fiducial probe binding sequence (catcaacgccacgatcagct), and (4) a unique pair of index sequences at the 5′ (ACGTCCGCCGCATCTACGAG) and 3′ (CTGAACGGCGCACGTGTCTT) ends that allowed this probe set to be specifically amplified from a larger oligopool ordered from CustomArray. The probe set was synthesized from the oligopool as in the work by Mateo and colleagues [41], using forward primer (ACGTCCGCCGCATCTACGAG) and reverse primer (AAGACACGTGCGCCGTTCAG). Oligopaint probe sequences are provided in S2 Data; readout probe sequences and strand-displacement oligo sequences are provided in S1 Table and S2 Table.

### Sequential immuno-FISH experiments

Gonads from animals heterozygous for *mIn1* were dissected and spread on a coverslip using partial spreading conditions. After the sample was dried, it was incubated in −20°C methanol for 20 minutes. Then, the sample was rehydrated by washing in PBST for 5 minutes each. Next, the sample was incubated in 0.1 M HCl for 5 minutes and washed in PBST 3 times for 5 minutes each. The samples were then incubated for 5 minutes each in increasing concentrations of formamide in 2x SSCT: 5%, 10%, 25%, and 50%. The sample was then incubated in a prewarmed 42°C solution of 50% formamide in 2x SSCT for 1 hour. 2 μL of Oligopaint probe (1,000 ng/μL in dH2O) was diluted into 30 μL of hybridization solution (50% formamide, 10% Dextran Sulfate, 2x SSC, 0.1% Tween-20) for each coverslip. After 1 hour incubation, slides were taken out of the 50% formamide solution, wiped, and incubated in 95% ethanol for 5 minutes. Then, the probe hybridization solution was applied to the sample with a slide, and the sample was denatured for 10 minutes at 90°C on a heat block. After denaturing, the sample was incubated with the probe hybridization solution at 42°C overnight. The next day, samples were washed 2 times in 42°C 50% formamide in 2x SSCT for 30 minutes each, and the slide was removed from the coverslip. Then, the sample was incubated for 5 minutes each in solutions with decreasing concentrations of formamide in 2x SSCT: 25%, 10%, and 5%. Samples were then washed 2 times for 10 minutes each in 2x SSCT and postfixed in 2% formaldehyde and 8% glutaraldehyde in 1x PBS followed by three 5 minute washes in PBST. The sample was blocked in 1% w/v BSA in PBST at room temperature and further incubated overnight with a primary antibody against HTP-3 at room temperature (antibodies diluted in 1% w/v BSA in PBST supplied with 0.05% w/v NaN_3_). Coverslips were washed 3 times for 5 minutes in PBST and blocked again in 1% w/v BSA in PBST. Coverslips were incubated with a secondary antibody conjugated to Alexa 488 for 2 hours at room temperature and subsequently washed 3 times for 5 minutes in PBST, and samples were covered in Vectashield. A homebuilt microfluidic chamber was mounted onto to the coverslip with silicon, and this assembly was mounted onto a homebuilt holder for imaging using the OMX Blaze system.

In order to visualize the hybridization probes in 10 sequential 100 kbp steps, readout oligos (which hybridize to barcode sequences specific to each 10 kbp in the oligopaint library) were pooled in groups of 10 for each hybridization step, and readout probes were labeled indirectly via hybridization with Cy5-marked “imaging” oligos (Cy5-5p-TGGGACGGTTCCAATCGGATC). A fiducial probe, which labels the entire 1 Mbp region, was marked by Cy3. Sequential hybridization of readout and imaging oligos and probe displacement steps were performed as in the work by Mateo and colleagues [41]. At each hybridization step, wide-field image stacks of midpachytene nuclei (in 50% Vectashield in 1x SCC) were acquired for all 3 channels. A 3D-SIM image of the HTP-3 and fiducial signals was acquired after the fifth round of hybridization. Wide-field image stacks were deconvolved and corrected for drift in 3D with ImageJ Plugin: “Correct 3D drift” using first the fiducial probe signal and then HTP-3 as a reference channel. Images were SUM-intensity projected and doubled in size to fit the 3D-SIM image size. Brightness differences between individual hybridization/imaging steps were corrected with ImageJ Bleach Correction (using “Histogram matching”) in all 3 channels. Chromosome axes for which fiducial probes were unambiguously assignable were cropped individually from 3D-SIM images and overlaid with wide-field HTP-3 signals in the SUM projected pictures. When axes were imaged from a frontal view; i.e., when 2 roughly parallel axis signals could be detected, the fiducial probe signal was inferred to correspond to DNA associated with the closer of the 2 axis signals. Only cases in which an unambiguous assignment could be made were analyzed. Individual signals for each hybridization step that fully or partially overlapped with HTP-3 signals were considered “axis-associated.” Signals not colocalizing with HTP-3 were considered to be in “loops.” Brightness and contrast were adjusted for display.

## Supporting information

S1 Fig Requirement for HTP-3 in organizing cohesins into axial arrays. (A) SIM image of a fully spread nucleus stained for REC-8::GFP, SMC-1, and HTP-3. (B) Deconvolved wide-field mages of partially spread nuclei from a wild-type gonad (top) or *htp-3* mutant gonads (bottom) immunostained for SC central region protein SYP-1 and either SMC-1 (which represents all meiotic cohesin complexes present) or REC-8 (which represents a subset of meiotic cohesin complexes). In the *htp-*3 mutant, SMC-1 and REC-8 were not detected as linear arrays of foci or as continuous axes in either early or late prophase nuclei (indicated by the presence of 1–3 SYP-1 aggregates). Although it was reported previously that REC-8 is not loaded onto chromosomes in *htp-3* mutants, the sample preparation method used here releases soluble proteins, thereby enabling detection of residual chromosome-bound proteins that can be difficult to detect in whole mount preparations. SC, synaptonemal complex; SIM, structured illumination microscopy.
(TIF)

S2 Fig Structure and features of ExChrs. (A) Circular structure of ExChrs. (Left) Schematic illustrating that multiple circular and/or linear DNA molecules co-injected into the *C*. *elegans* germ line co-assemble into extrachromosomal arrays that can be inherited as minichromosomes. (Right) SIM images of HTP-3-marked chromosome axes in spread nuclei carrying ExChrs, which appear as rings. Insets show multiple rings cropped from different nuclei from the same strain; the fact that rings from a given strain are similar in size indicates that, once formed, ExChrs tend to be stable in size. The rings depicted are 2 different examples from 21 independent lines generated using the same selection scheme [*cb-unc-119(+)* in the *unc-119(ed3)* mutant], either alone or together with 10% to 90% *E*. *coli* genomic DNA (linearized by sonication and size selected to 1–5 kbp by gel extraction) in the injection mix. The transmission rates of the Unc+ trait in self-fertilizing hermaphrodites varied widely, ranging from 10% to 95% and did not correlate with the percentage of *E*. *coli* DNA present in the injection mix. In 18 of the 21 lines, ExChrs could be detected as additional HTP-3-positive structures in meiotic prophase nuclei, and in 16 of these 18 cases, the ExChrs were reliably resolvable as rings by SIM in partially spread gonads; as the 2 ExChrs that were not verified as rings were the smallest of the 18, we speculate that they may also be rings, but their small size prevented their ring structure from being resolved microscopically. (B) Density of axis components on circular ExChrs. (Top) For each of the 2 ExChr-bearing strains depicted, the large images on the right show portions of nuclei from partially spread gonads prepared using conditions that preserve SCs, stained for HTP-3, COH-3/4, and SYP-1. At the left, schematic representations of the DNA composition of *meEx001* and *oxEx1578* are presented. (Bottom) Quantification of axis foci on ExChrs in fully spread nuclei stained for either REC-8 or COH-3/4 together with HTP-3. In the graphs, each data point represents a single ExChr, with the X-axis indicating the numbers of REC-8 or COH-3/4 foci and the Y-axis indicating the numbers of HTP-3 foci counted on that ExChr. All data acquired are presented; plotted values are available in S1 Data. For both ExChrs examined, the numbers of HTP-3 and REC-8 or COH-3/4 foci are strongly correlated (*p* < 0.001), and the slopes of the linear regression lines are near to 1 (for *meEx001* REC-8 versus HTP-3, r = 0.85, slope = 0.79; for *meEx001* COH-3/4 versus HTP-3, r = 0.88, slope = 0.84; for *oxEx1578* REC-8 versus HTP-3, r = 0.94, slope = 0.76; for *oxEx1578* COH-3/4 versus HTP-3, r = 0.76, slope = 0.79). Further, in 3 of the 4 cases, a line with slope = 1 falls within the 95% confidence interval for the slope of the linear regression line (indicated by the gray shaded area), consistent with a near 1:1:1 correspondence between HORMAD, REC-8, and COH-3/4 foci, as observed for spread chromosome segments (depicted in Fig 2). r values, *p* values, slopes, and 95% confidence intervals for the slopes were calculated and plotted using GraphPad Prism software. Thus, we infer that axis organization on the ExChrs is similar to that observed for normal chromosomes. ExChr, extrachromosomal array; SC, synaptonemal complex; SIM, structured illumination microscopy.
(TIF)

S3 Fig Functional distinctions between REC-8 and COH-3/4 cohesin complexes. (A) SIM images of HORMAD proteins and REC-8 in partially spread meiotic prophase nuclei from the *coh-3 coh-4* mutant, illustrating the requirement for COH-3/4 in axis organization. HORMADs and REC-8 still bind to chromatin in this mutant, but the high degree of colocalization between HTP-3, HTP-1/2, and REC-8 seen in the WT is not observed, and continuous axes do not form. (B) Reduction in chromosome-associated REC-8 cohesion-conferring complexes occurs independently of installation of COH-3/4 axis-organizing cohesins. Nonspread gonads from WT, *coh-3/4*, and *wapl-1* worms, with relative intensities of REC-8 immunofluorescence signals illustrated using the indicated color scale. The drop of REC-8 levels observed in the WT does not depend on the loading of COH-3/4, as it still occurs in their absence. However, a drop in REC-8 levels does not occur in the *wapl-1* mutant. (C) SIM images of individual chromosomes from partially spread meiotic prophase nuclei prepared using conditions that preserve association of SC central region proteins and maintain inter-axis distances comparable to in situ preparations [57]. (Left) Representative example images of SCs in a WT pachytene nucleus, provided together with traces of the paths of individual SCs that were generated using the ImageJ plugin “Simple Neurite Tracer” [56], illustrating that the paths of all 6 individual synapsed chromosome pairs can be reliably traced in 3D using this approach. (Right) Example images of SCs of straightened chromosomes, indicating the average lengths of wild-type early pachytene SCs (identified based on the presence of multiple recombination foci [marked by RPA and BLM] per SC), wild-type late pachytene SCs (which have a single bright COSA-1-marked crossover site focus per SC), and the intersister SCs that are formed in a *rec-8* mutant. SC, synaptonemal complex; SIM, structured illumination microscopy.
(TIF)

S4 Fig Supporting evidence for quantification of cohesin molecules. (A) SIM images of fully spread nuclei from worms expressing both REC-8::GFP and REC-8::3xFLAG, immunostained for HTP-3, FLAG, and GFP using sequential immunostaining protocols. (Top) Anti-FLAG was applied first, followed by secondary detection of FLAG, before primary and secondary immunodetection of HTP-3 and GFP. (Bottom) Anti-GFP was applied first, followed by secondary detection of GFP, before primary and secondary immunodetection of HTP-3 and FLAG. In all 3 orders in which the experiment was performed (sequential staining in both directions and simultaneous co-staining with all primary antibodies as in Fig 3A), GFP and FLAG signals both localize to chromosome axes (marked by HTP-3) in similar numbers, but most GFP and FLAG signals do not colocalize. Middle panels were generated using ImageJ plugin “ColocThreshold” with white indicating the infrequent pixels with significant FLAG and GFP colocalization (colocalizing pixels are in white, noncolocalizing pixels are in red and green); contrast and brightness were adjusted for display to improve visibility of the white pixels. (B) Similar immunofluorescence signals for purified GFP protein and chromosome-associated REC-8::GFP foci. Purified GFP protein was spotted onto a glass slide, and nuclei from worms expressing REC-8::GFP (as the sole source of REC-8) were spread and fixed on top. Images are SUM-intensity projections of immunofluorescence detection of GFP with a mouse monoclonal antibody (left) or with a rabbit polyclonal antibody (center, right); images at the right are from a control slide without spotted GFP protein. Corresponding graphs plot background-subtracted fluorescence values for 4 × 4 pixel ROIs encompassing single REC-8::GFP foci (orange) or purified GFP protein foci (blue) from the same image. (C) COH-3/4 and SMC-1 fluorescence values correlate with each other in fully spread nuclei from worms expressing SMC-1::HA. (Left) SIM image of well-spread chromosome segments in which relative intensities of immunofluorescence signals are depicted using the indicated color scale, illustrating the strong similarity between the fluorescence intensity patterns for COH-3/4 (top) and SMC-1::HA (bottom). (Right) Graph plotting fluorescence values for SMC-1::HA (x-axis) and COH-3/4 (Y-axis) in 800 × 800 nm ROIs centered on individual axis foci from SUM projected, wide-field images. R^2^ value reflects a strong positive correlation between SMC-1 and COH-3/4 fluorescence values in individual foci. Plotted values for panels B and C are available in S1 Data. ROI, region of interest; SIM, structured illumination microscopy.
(TIF)

S5 Fig Assessment of relative levels of chromosome associated HORMAD proteins in whole nuclei. (A, left) SUM-projected nondeconvolved wide-field images (left) of leptotene nuclei from partially spread gonads from worms expressing either 3xFLAG::HTP-3::GFP or GFP::HIM-3, processed in parallel for anti-GFP immunofluorescence on the same slide. Each data point in the graph (right) represents the total fluorescence in a single nucleus. (Right) SUM-projected nondeconvolved wide-field images of late pachytene nuclei from partially spread gonads from worms expressing either REC-8::3xFLAG and GFP::COSA-1 or 3xFLAG::HTP-3::GFP, processed in parallel for anti-FLAG immunofluorescence on the same slide; native GFP fluorescence was used to distinguish the genotypes of the gonads analyzed. Each data point in the graph represents the total fluorescence in a single nucleus; data are normalized to Average REC-8::FLAG fluorescence = 1. (B) Fully spread nuclei from worms expressing 3xFLAG::HTP-3::GFP and from worms expressing HTP-1::GFP (either in the presence or absence of HTP-2) were prepared as in Fig 3D on the same slide (HTP-2 is present in the example image on the left). Nuclei were stained sequentially for GFP and COH-3/4, followed by detection of the FLAG epitope to identify the nuclei expressing 3xFLAG::HTP-3::GFP. Total immunofluorescence signals for GFP (green channel) and COH-3/4 (far-red channel) were measured in ROIs drawn around each individual nucleus. Each data point in the graph represents the ratio between total GFP signal and total COH-3/4 signal for a single nucleus (to account for differences in the degree of spreading of individual nuclei). Data for the 2 experiments were normalized to each other using the mean value for HTP-3::GFP as a normalization standard. In the graph, HTP-3::GFP values from the first experimental setup (presence of HTP-2 in both genotypes) are represented. (C) SUM-intensity projections of nonspread WT gonads stained for DAPI, HTP-3, and HTP-1/2 or DAPI, HTP-3, and HIM-3, and the gonads were divided as depicted into 19 equal-sized, half-overlapping ROIs from the mitotic tip to start of cellularization (late diplotene). For each HORMAD being evaluated (HTP-3, HIM-3, or HTP-1/2), the ratio between total immunofluorescence signal and total DAPI signal was determined for each ROI. For the plots of relative fluorescence (for each HORMAD) over the course of meiotic prophase progression, measured values were normalized to the average value for the set of ROIs spanning from the onset of meiotic prophase (ROI 6) through the end of the pachytene stage (ROI 16–17). Two gonads were averaged for each staining. Note that HTP-3 levels remain stable over the course of meiotic prophase, whereas HTP-1/2 and HIM-3 levels increase. Plotted values for panels A, B, and C are available in S1 Data. ROI, region of interest.
(TIF)

S6 Fig Display of individual hybridization sequences allowing inferences of DNA loop paths. Each panel depicts a sequence of images (from left to right) from iterative FISH experiments in which consecutive 100 kbp DNA segments were individually visualized in red (using pools of secondary readout oligos), and the full 1 Mbp probed region was visualized in blue (fiducial probe). Panels 1–30, 32, 33, and 38 depict a subset of chromosome segments of the analyzed data set that met the following criteria: (1) the 2 sister-chromatid signals could be resolved for at least every other step, and (2) the fiducial probe signal could be assigned unambiguously to a chromosome axis visualized by HTP-3 immunofluorescence. For reference, a dashed white line in the initial frame of each panel indicates the path of the HTP-3 axis signal considered for analysis. When axes were imaged from a frontal perspective, i.e., when 2 roughly parallel axis signals could be detected, the fiducial probe signal was inferred to correspond to DNA associated with the closer of the 2 axis signals. Ambiguous signals (which can represent the majority in a given microscopic field) were not analyzed. Panels 1 and 2 correspond to 2 different portions of the same chromosome. For a few individual hybridization steps in panels 7, 8, 24, and 27, complex fluorescence signals were co-assigned to a given chromatid based on proximity. Panel 4 includes one ambiguous step (step 2) that can be interpreted differently than displayed. Panels 31, 34–37, and 39 depict chromosome segments that include 2 or more consecutive unresolved sister-chromatid signals. On the left, the consecutive 100 kbp DNA segments represented in each left-to-right image panel are schematically represented from top to bottom. Positions where FISH signals overlap with the axis signal (A) are indicated in the center column in gray or black cells, whereas positions where FISH signals are in loops >100 kbp in size (L) are indicated in red cells flanking the center column. Unresolvable signals that colocalized with the assigned chromosome axis are represented as “AA” (114 cases in the displayed data set). If signals were resolvable, but both colocalize with the assigned chromosome axis, they are represented as “A__A” (7 cases). If signals were not resolvable but did not colocalize with the chromosome axis, they are represented as LL (4 cases, dark red fields). For the first 7 chromosome segments analyzed, curves illustrating potential paths of DNA loops that are larger than 100 kbp (i.e., where one or several consecutive 100 kbp DNA segments were scored as “L”) are overlaid on the schematic diagrams. In this representation, loops from 2 different sister chromatids are indicated by solid and dashed lines, respectively. In some cases, e.g., panel 3, only one set of potential paths is compatible with the underlying diagram and corresponding images. For other cases, alternative paths are possible; the maximum-sized potential loop paths are indicated in blue, minimum-sized potential loop paths in green. We also analyzed the full 10-step FISH sequences for a total of 26 chromosomes. This unbiased data set was used for Fig 4C and 4D and the corresponding statistical analyses presented in the text. Notably, LL signals represented only a small fraction (3.5%) of the total signals in this unbiased data set; thus, the conclusion that symmetric loops >100 kbp in size are infrequent would not be affected by the possibility that unresolved LL signals might be underrepresented among the displayed images. FISH, fluorescence in situ hybridization.
(TIF)

S7 Fig Testing asymmetry of sister-chromatid loops in minimally disrupted tissue. (A) Illustration indicating which side of the nucleus was assayed to determine the distances between FISH signal junctions and the chromosome axis for the analysis presented in panel B. The side of the nucleus that adheres to the glass (magenta frame, left) in the partial spreading procedure is visibly more flattened than the nonadhering side (green frame, right). Moreover, adherence of chromatin/DNA to the glass slide itself may potentially introduce artifacts affecting DNA/chromatin organization. Therefore, only FISH signals that were associated with chromosome segments in the nonadhering side of the nucleus were assayed. (B) Immuno-FISH evidence for asymmetry between sister-chromatid loops in minimally disrupted tissue in 3D. *mIn1*, a rearranged version of chromosome II harboring an 8.2 Mbp internal inversion was used for assessment of sister-chromatid loop relationships. *mIn1* heterozygosity results in wide separation between the FISH signals for the normal and rearranged homologs, thereby enabling assessment of sister-chromatid signals. Images show 2 examples (early pachytene, left and late pachytene, right) of single Z planes in which the 200 kbp FISH signals (chromosome II 11.5–11.7 Mbp) for both homologs could be found in the same Z-plane. FISH signals were only assessed if they were visible as a single focus or 2 foci; splintered signals were excluded. The distance between the centroid of each FISH signal and the center of the nearest axis signal (visualized by HTP-3 immunostaining) was measured in 3D; plotted values are available in S1 Data. A signal was classified as “A” if its centroid was located <240 nm (<3 pixel in XY) from the center of the nearest axis segment and classified as “L” if located farther away. FISH, fluorescence in situ hybridization.
(TIF)

S1 Data Numerical values underlying graphs. This file contains all data used to generate the graphs presented in the main figures and S2 Fig, S3 Fig, S4 Fig, S5 Fig and S7 Fig, compiled as an Excel (.xlsx) file. Notes relevant to specific data sets are included as text boxes on the appropriate sheets.
(XLSX)

S2 Data Oligopaint probes. This file contains Oligopaint Probe Sequences as a.txt file.
(TXT)

S1 Table Readout probes. This file contains Readout probe sequences as an Excel (.xlsx) file.
(XLSX)

S2 Table Strand-displacement oligos. This file contains Strand-displacement oligo sequences as an Excel (.xlsx) file.
(XLSX)

## Abbreviations

DSB: double strand break
ES cells: embryonic stem cells
ExChr: extrachromosomal array
FCS: fluorescence correlation spectroscopy
FISH: fluorescence in situ hybridization
ROI: region of interest
SC: synaptonemal complex
SIM: structured illumination microscopy
WT: wild type
