# Supplementary figures and images for: Quantitative cytogenetics reveals molecular stoichiometry and longitudinal organization of meiotic chromosome axes and loops
Source: PLoS Biol. 2020 Aug 19;18(8):e3000817. doi: 10.1371/journal.pbio.3000817 (PMC7458323; doi:10.1371/journal.pbio.3000817)

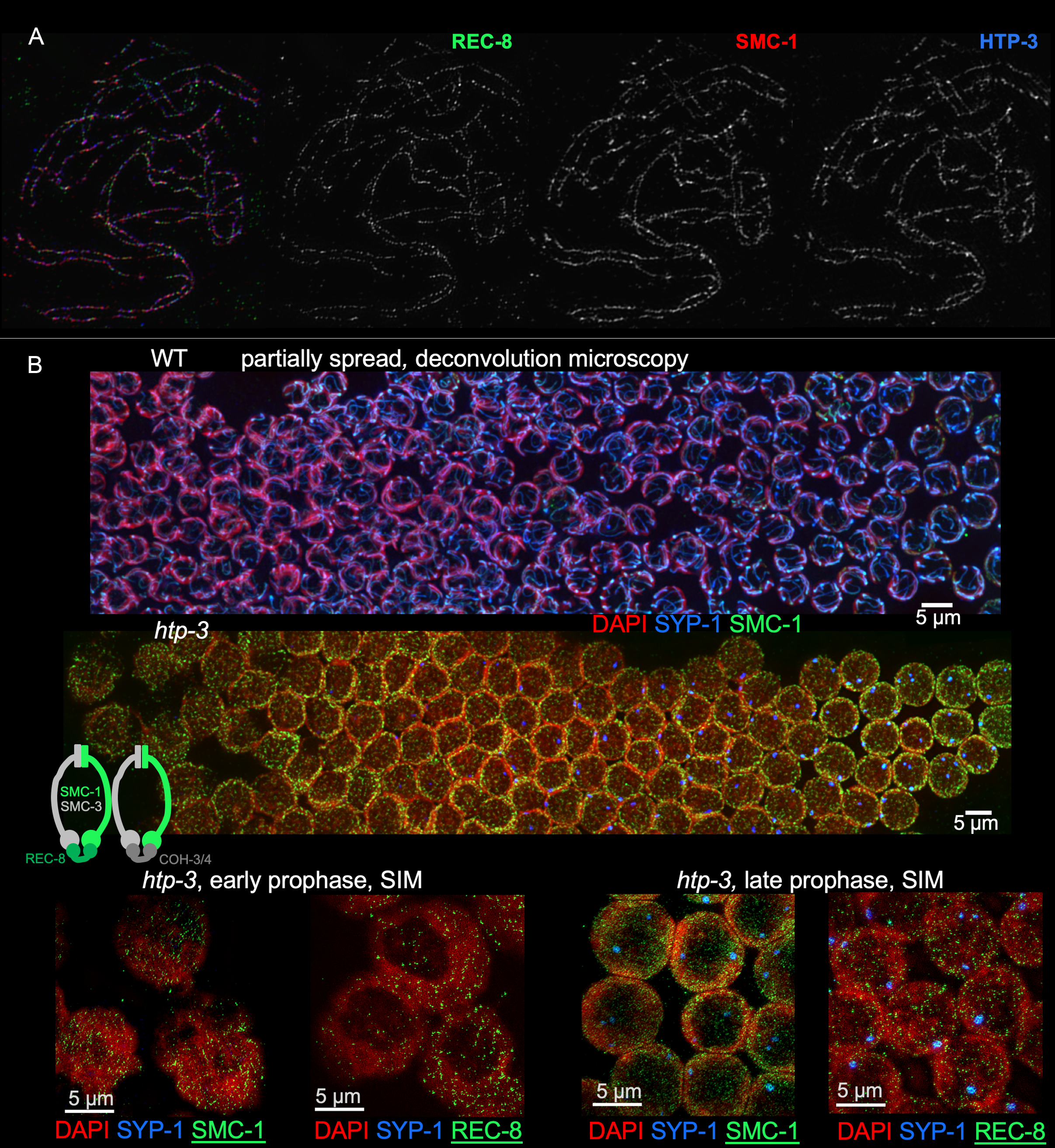

Supplement: S1 Fig — (A) SIM image of a fully spread nucleus stained for REC-8::GFP, SMC-1, and HTP-3. (B) Deconvolved wide-field mages of partially spread nuclei from a wild-type gonad (top) or htp-3 mutant gonads (bottom) immunostained for SC central region protein SYP-1 and either SMC-1 (which represents all meiotic cohesin complexes present) or REC-8 (which represents a subset of meiotic cohesin complexes). In the htp-3 mutant, SMC-1 and REC-8 were not detected as linear arrays of foci or as continuous axes in either early or late prophase nuclei (indicated by the presence of 1–3 SYP-1 aggregates). Although it was reported previously that REC-8 is not loaded onto chromosomes in htp-3 mutants, the sample preparation method used here releases soluble proteins, thereby enabling detection of residual chromosome-bound proteins that can be difficult to detect in whole mount preparations. SC, synaptonemal complex; SIM, structured illumination microscopy. (TIF) [file pbio.3000817.s001.tif]

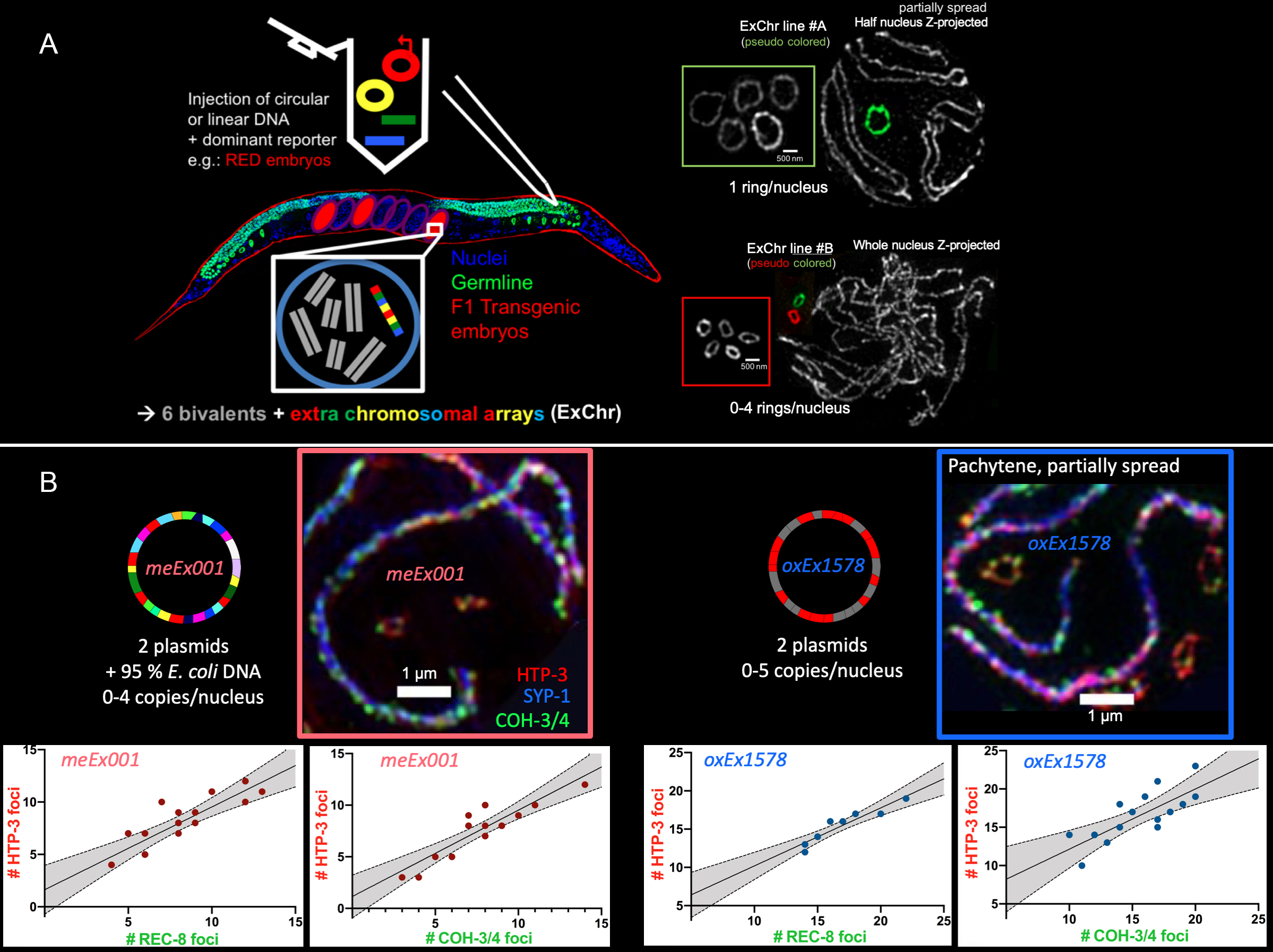

Supplement: S2 Fig — (A) Circular structure of ExChrs. (Left) Schematic illustrating that multiple circular and/or linear DNA molecules co-injected into the C. elegans germ line co-assemble into extrachromosomal arrays that can be inherited as minichromosomes. (Right) SIM images of HTP-3-marked chromosome axes in spread nuclei carrying ExChrs, which appear as rings. Insets show multiple rings cropped from different nuclei from the same strain; the fact that rings from a given strain are similar in size indicates that, once formed, ExChrs tend to be stable in size. The rings depicted are 2 different examples from 21 independent lines generated using the same selection scheme [cb-unc-119(+) in the unc-119(ed3) mutant], either alone or together with 10% to 90% E. coli genomic DNA (linearized by sonication and size selected to 1–5 kbp by gel extraction) in the injection mix. The transmission rates of the Unc+ trait in self-fertilizing hermaphrodites varied widely, ranging from 10% to 95% and did not correlate with the percentage of E. coli DNA present in the injection mix. In 18 of the 21 lines, ExChrs could be detected as additional HTP-3-positive structures in meiotic prophase nuclei, and in 16 of these 18 cases, the ExChrs were reliably resolvable as rings by SIM in partially spread gonads; as the 2 ExChrs that were not verified as rings were the smallest of the 18, we speculate that they may also be rings, but their small size prevented their ring structure from being resolved microscopically. (B) Density of axis components on circular ExChrs. (Top) For each of the 2 ExChr-bearing strains depicted, the large images on the right show portions of nuclei from partially spread gonads prepared using conditions that preserve SCs, stained for HTP-3, COH-3/4, and SYP-1. At the left, schematic representations of the DNA composition of meEx001 and oxEx1578 are presented. (Bottom) Quantification of axis foci on ExChrs in fully spread nuclei stained for either REC-8 or COH-3/4 together with HTP-3. [file pbio.3000817.s002.tif]

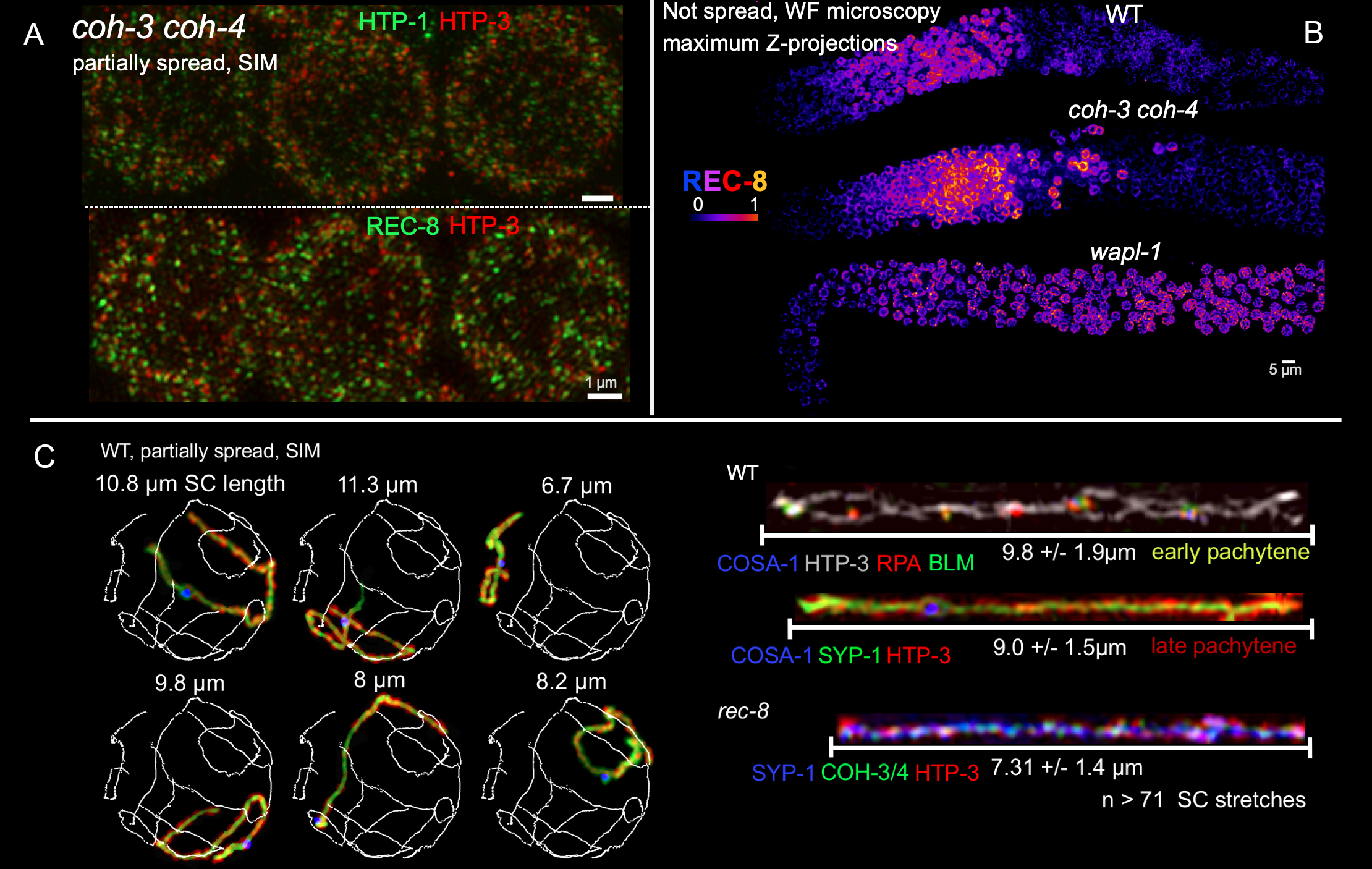

Supplement: S3 Fig — (A) SIM images of HORMAD proteins and REC-8 in partially spread meiotic prophase nuclei from the coh-3 coh-4 mutant, illustrating the requirement for COH-3/4 in axis organization. HORMADs and REC-8 still bind to chromatin in this mutant, but the high degree of colocalization between HTP-3, HTP-1/2, and REC-8 seen in the WT is not observed, and continuous axes do not form. (B) Reduction in chromosome-associated REC-8 cohesion-conferring complexes occurs independently of installation of COH-3/4 axis-organizing cohesins. Nonspread gonads from WT, coh-3/4, and wapl-1 worms, with relative intensities of REC-8 immunofluorescence signals illustrated using the indicated color scale. The drop of REC-8 levels observed in the WT does not depend on the loading of COH-3/4, as it still occurs in their absence. However, a drop in REC-8 levels does not occur in the wapl-1 mutant. (C) SIM images of individual chromosomes from partially spread meiotic prophase nuclei prepared using conditions that preserve association of SC central region proteins and maintain inter-axis distances comparable to in situ preparations [57]. (Left) Representative example images of SCs in a WT pachytene nucleus, provided together with traces of the paths of individual SCs that were generated using the ImageJ plugin “Simple Neurite Tracer” [56], illustrating that the paths of all 6 individual synapsed chromosome pairs can be reliably traced in 3D using this approach. (Right) Example images of SCs of straightened chromosomes, indicating the average lengths of wild-type early pachytene SCs (identified based on the presence of multiple recombination foci [marked by RPA and BLM] per SC), wild-type late pachytene SCs (which have a single bright COSA-1-marked crossover site focus per SC), and the intersister SCs that are formed in a rec-8 mutant. SC, synaptonemal complex; SIM, structured illumination microscopy. (TIF) [file pbio.3000817.s003.tif]

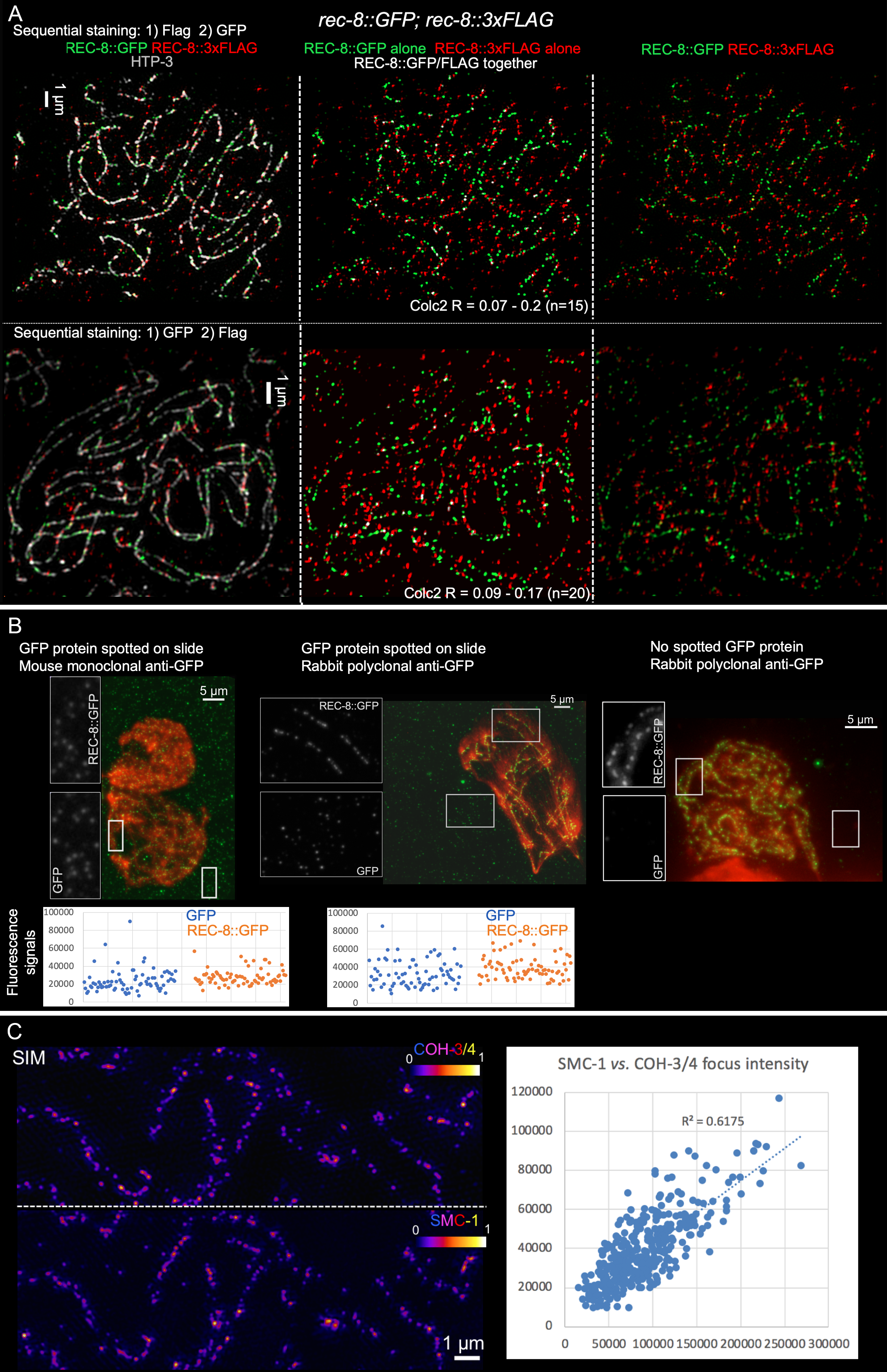

Supplement: S4 Fig — (A) SIM images of fully spread nuclei from worms expressing both REC-8::GFP and REC-8::3xFLAG, immunostained for HTP-3, FLAG, and GFP using sequential immunostaining protocols. (Top) Anti-FLAG was applied first, followed by secondary detection of FLAG, before primary and secondary immunodetection of HTP-3 and GFP. (Bottom) Anti-GFP was applied first, followed by secondary detection of GFP, before primary and secondary immunodetection of HTP-3 and FLAG. In all 3 orders in which the experiment was performed (sequential staining in both directions and simultaneous co-staining with all primary antibodies as in Fig 3A), GFP and FLAG signals both localize to chromosome axes (marked by HTP-3) in similar numbers, but most GFP and FLAG signals do not colocalize. Middle panels were generated using ImageJ plugin “ColocThreshold” with white indicating the infrequent pixels with significant FLAG and GFP colocalization (colocalizing pixels are in white, noncolocalizing pixels are in red and green); contrast and brightness were adjusted for display to improve visibility of the white pixels. (B) Similar immunofluorescence signals for purified GFP protein and chromosome-associated REC-8::GFP foci. Purified GFP protein was spotted onto a glass slide, and nuclei from worms expressing REC-8::GFP (as the sole source of REC-8) were spread and fixed on top. Images are SUM-intensity projections of immunofluorescence detection of GFP with a mouse monoclonal antibody (left) or with a rabbit polyclonal antibody (center, right); images at the right are from a control slide without spotted GFP protein. Corresponding graphs plot background-subtracted fluorescence values for 4 × 4 pixel ROIs encompassing single REC-8::GFP foci (orange) or purified GFP protein foci (blue) from the same image. (C) COH-3/4 and SMC-1 fluorescence values correlate with each other in fully spread nuclei from worms expressing SMC-1::HA. (Left) SIM image of well-spread chromosome segments in which relative intensities of [file pbio.3000817.s004.tif]

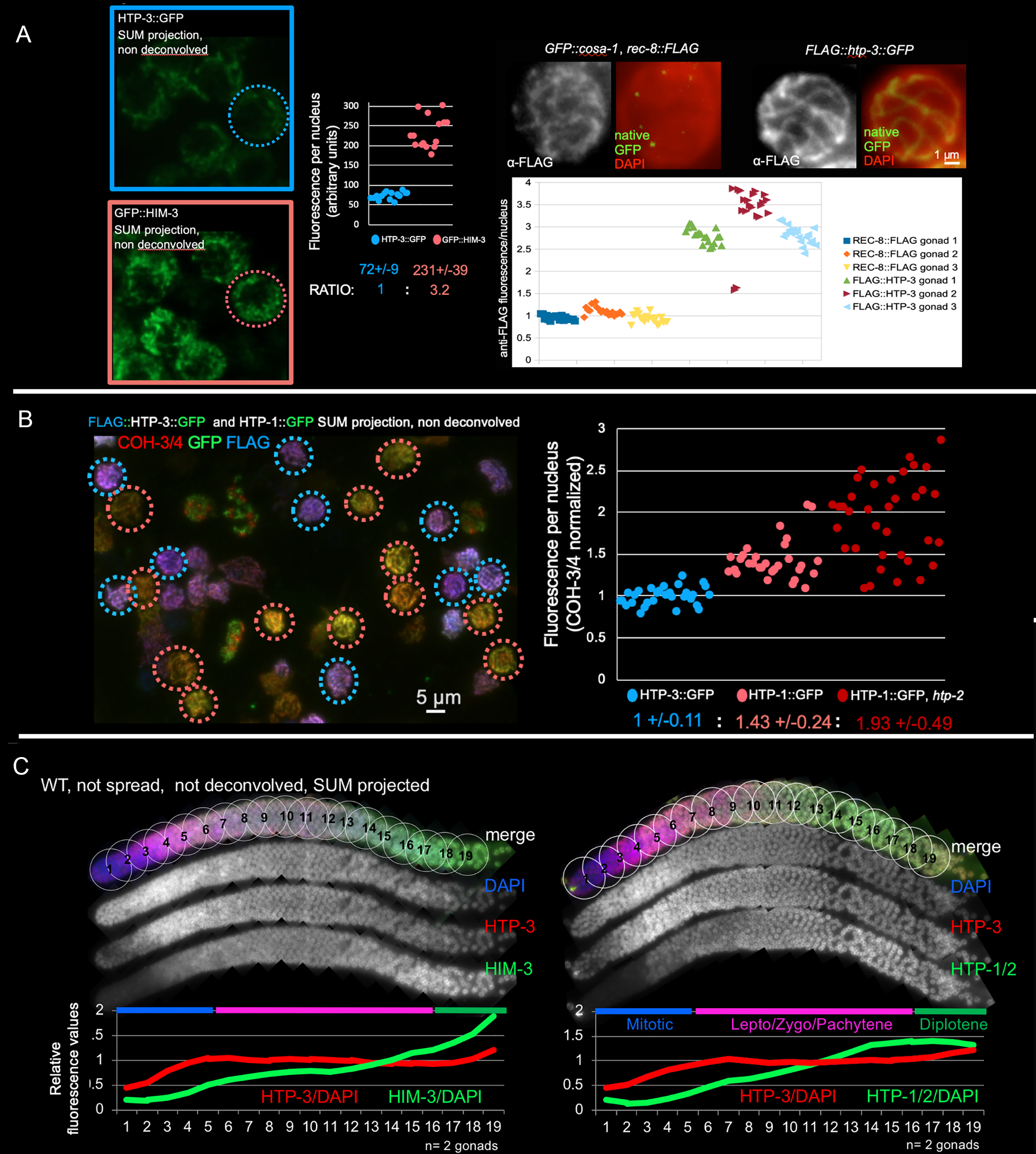

Supplement: S5 Fig — (A, left) SUM-projected nondeconvolved wide-field images (left) of leptotene nuclei from partially spread gonads from worms expressing either 3xFLAG::HTP-3::GFP or GFP::HIM-3, processed in parallel for anti-GFP immunofluorescence on the same slide. Each data point in the graph (right) represents the total fluorescence in a single nucleus. (Right) SUM-projected nondeconvolved wide-field images of late pachytene nuclei from partially spread gonads from worms expressing either REC-8::3xFLAG and GFP::COSA-1 or 3xFLAG::HTP-3::GFP, processed in parallel for anti-FLAG immunofluorescence on the same slide; native GFP fluorescence was used to distinguish the genotypes of the gonads analyzed. Each data point in the graph represents the total fluorescence in a single nucleus; data are normalized to Average REC-8::FLAG fluorescence = 1. (B) Fully spread nuclei from worms expressing 3xFLAG::HTP-3::GFP and from worms expressing HTP-1::GFP (either in the presence or absence of HTP-2) were prepared as in Fig 3D on the same slide (HTP-2 is present in the example image on the left). Nuclei were stained sequentially for GFP and COH-3/4, followed by detection of the FLAG epitope to identify the nuclei expressing 3xFLAG::HTP-3::GFP. Total immunofluorescence signals for GFP (green channel) and COH-3/4 (far-red channel) were measured in ROIs drawn around each individual nucleus. Each data point in the graph represents the ratio between total GFP signal and total COH-3/4 signal for a single nucleus (to account for differences in the degree of spreading of individual nuclei). Data for the 2 experiments were normalized to each other using the mean value for HTP-3::GFP as a normalization standard. In the graph, HTP-3::GFP values from the first experimental setup (presence of HTP-2 in both genotypes) are represented. (C) SUM-intensity projections of nonspread WT gonads stained for DAPI, HTP-3, and HTP-1/2 or DAPI, HTP-3, and HIM-3, and the gonads were divided as depicted into 19 equal-sized, h [file pbio.3000817.s005.tif]

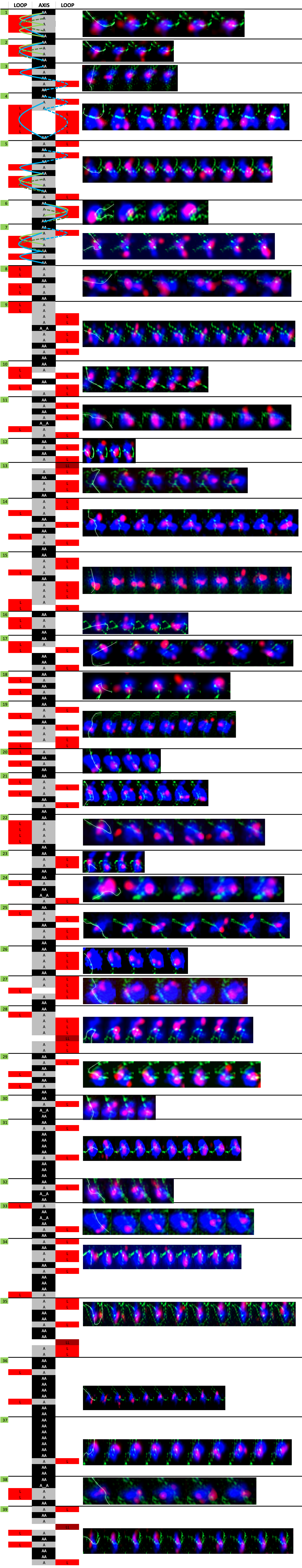

Supplement: S6 Fig — Each panel depicts a sequence of images (from left to right) from iterative FISH experiments in which consecutive 100 kbp DNA segments were individually visualized in red (using pools of secondary readout oligos), and the full 1 Mbp probed region was visualized in blue (fiducial probe). Panels 1–30, 32, 33, and 38 depict a subset of chromosome segments of the analyzed data set that met the following criteria: (1) the 2 sister-chromatid signals could be resolved for at least every other step, and (2) the fiducial probe signal could be assigned unambiguously to a chromosome axis visualized by HTP-3 immunofluorescence. For reference, a dashed white line in the initial frame of each panel indicates the path of the HTP-3 axis signal considered for analysis. When axes were imaged from a frontal perspective, i.e., when 2 roughly parallel axis signals could be detected, the fiducial probe signal was inferred to correspond to DNA associated with the closer of the 2 axis signals. Ambiguous signals (which can represent the majority in a given microscopic field) were not analyzed. Panels 1 and 2 correspond to 2 different portions of the same chromosome. For a few individual hybridization steps in panels 7, 8, 24, and 27, complex fluorescence signals were co-assigned to a given chromatid based on proximity. Panel 4 includes one ambiguous step (step 2) that can be interpreted differently than displayed. Panels 31, 34–37, and 39 depict chromosome segments that include 2 or more consecutive unresolved sister-chromatid signals. On the left, the consecutive 100 kbp DNA segments represented in each left-to-right image panel are schematically represented from top to bottom. Positions where FISH signals overlap with the axis signal (A) are indicated in the center column in gray or black cells, whereas positions where FISH signals are in loops >100 kbp in size (L) are indicated in red cells flanking the center column. Unresolvable signals that colocalized with the assigned chromosome axi [file pbio.3000817.s006.tif]

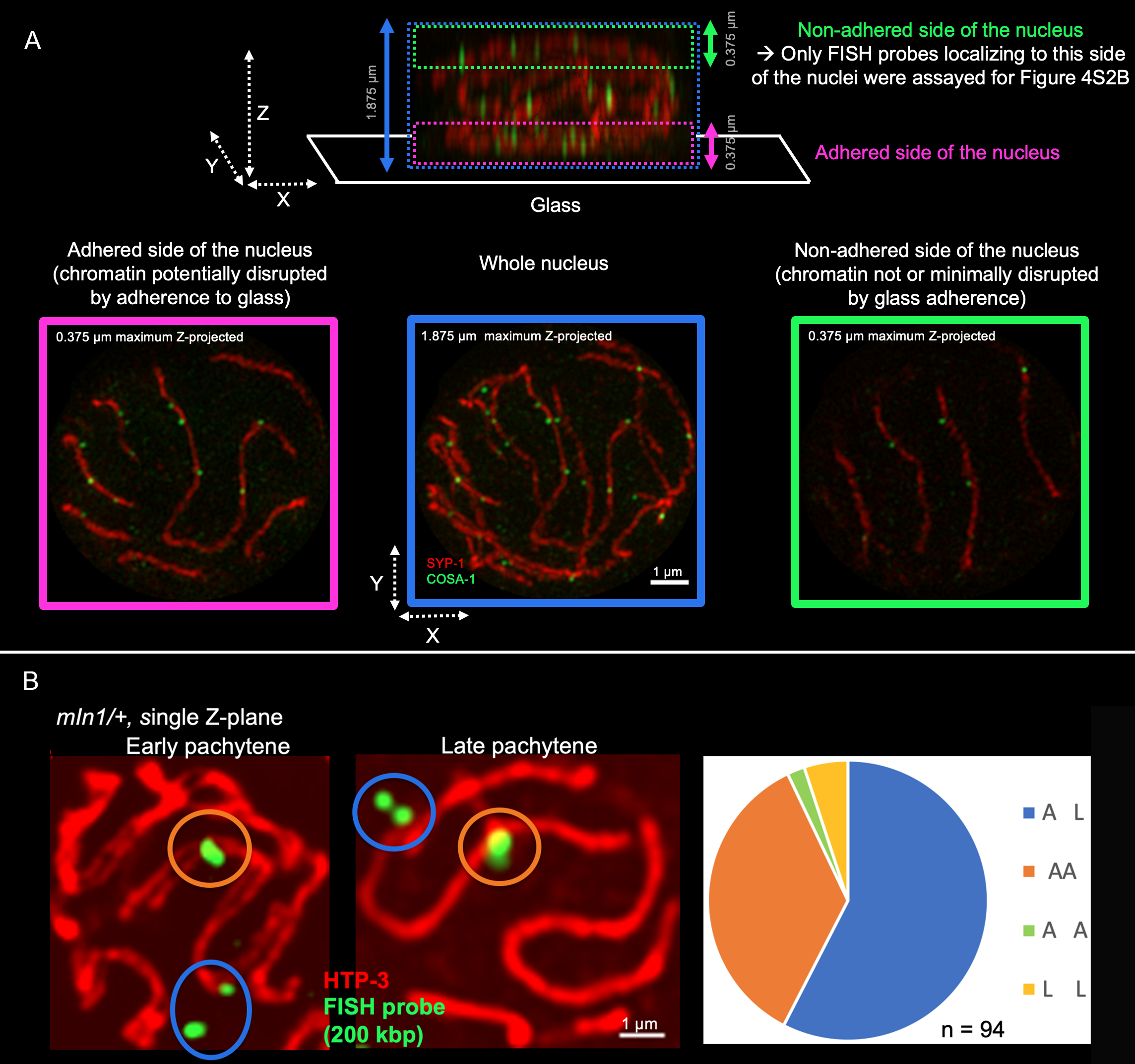

Supplement: S7 Fig — (A) Illustration indicating which side of the nucleus was assayed to determine the distances between FISH signal junctions and the chromosome axis for the analysis presented in panel B. The side of the nucleus that adheres to the glass (magenta frame, left) in the partial spreading procedure is visibly more flattened than the nonadhering side (green frame, right). Moreover, adherence of chromatin/DNA to the glass slide itself may potentially introduce artifacts affecting DNA/chromatin organization. Therefore, only FISH signals that were associated with chromosome segments in the nonadhering side of the nucleus were assayed. (B) Immuno-FISH evidence for asymmetry between sister-chromatid loops in minimally disrupted tissue in 3D. mIn1, a rearranged version of chromosome II harboring an 8.2 Mbp internal inversion was used for assessment of sister-chromatid loop relationships. mIn1 heterozygosity results in wide separation between the FISH signals for the normal and rearranged homologs, thereby enabling assessment of sister-chromatid signals. Images show 2 examples (early pachytene, left and late pachytene, right) of single Z planes in which the 200 kbp FISH signals (chromosome II 11.5–11.7 Mbp) for both homologs could be found in the same Z-plane. FISH signals were only assessed if they were visible as a single focus or 2 foci; splintered signals were excluded. The distance between the centroid of each FISH signal and the center of the nearest axis signal (visualized by HTP-3 immunostaining) was measured in 3D; plotted values are available in S1 Data. A signal was classified as “A” if its centroid was located <240 nm (<3 pixel in XY) from the center of the nearest axis segment and classified as “L” if located farther away. FISH, fluorescence in situ hybridization. (TIF) [file pbio.3000817.s007.tif]
